# Redirected nuclear glutamate dehydrogenase supplies Tet3 with α-ketoglutarate in neurons

**DOI:** 10.1038/s41467-021-24353-9

**Published:** 2021-07-02

**Authors:** Franziska R. Traube, Dilara Özdemir, Hanife Sahin, Constanze Scheel, Andrea F. Glück, Anna S. Geserich, Sabine Oganesian, Sarantos Kostidis, Katharina Iwan, René Rahimoff, Grazia Giorgio, Markus Müller, Fabio Spada, Martin Biel, Jürgen Cox, Martin Giera, Stylianos Michalakis, Thomas Carell

**Affiliations:** 1 Department of Chemistry, Ludwig-Maximilians-Universität München, Munich, Germany; 2 Department of Pharmacy – Center for Drug Research, Ludwig-Maximilians-Universität München, Munich, Germany; 3 Leiden University Medical Center, Center for Proteomics and Metabolomics, Leiden, The Netherlands; 4 Computational Systems Biochemistry, Max Planck Institute of Biochemistry, Martinsried, Germany; 5 Department of Ophthalmology, University Hospital, LMU Munich, Munich, Germany

**Keywords:** Neurochemistry, DNA methylation, Epigenetics and plasticity

## Abstract

Tet3 is the main α-ketoglutarate (αKG)-dependent dioxygenase in neurons that converts 5-methyl-dC into 5-hydroxymethyl-dC and further on to 5-formyl- and 5-carboxy-dC. Neurons possess high levels of 5-hydroxymethyl-dC that further increase during neural activity to establish transcriptional plasticity required for learning and memory functions. How αKG, which is mainly generated in mitochondria as an intermediate of the tricarboxylic acid cycle, is made available in the nucleus has remained an unresolved question in the connection between metabolism and epigenetics. We show that in neurons the mitochondrial enzyme glutamate dehydrogenase, which converts glutamate into αKG in an NAD^+^-dependent manner, is redirected to the nucleus by the αKG-consumer protein Tet3, suggesting on-site production of αKG. Further, glutamate dehydrogenase has a stimulatory effect on Tet3 demethylation activity in neurons, and neuronal activation increases the levels of αKG. Overall, the glutamate dehydrogenase-Tet3 interaction might have a role in epigenetic changes during neural plasticity.

## Abstract (web summary)

α-ketoglutarate (αKG) is an intermediate in the tricarboxylic acid cycle that is required in the nucleus for genomic DNA demethylation by Tet3. Here, the authors show that the enzyme glutamate dehydrogenase, which converts glutamate to αKG, is redirected from the mitochondria to the nucleus.

## Introduction

**Fig. 1:**
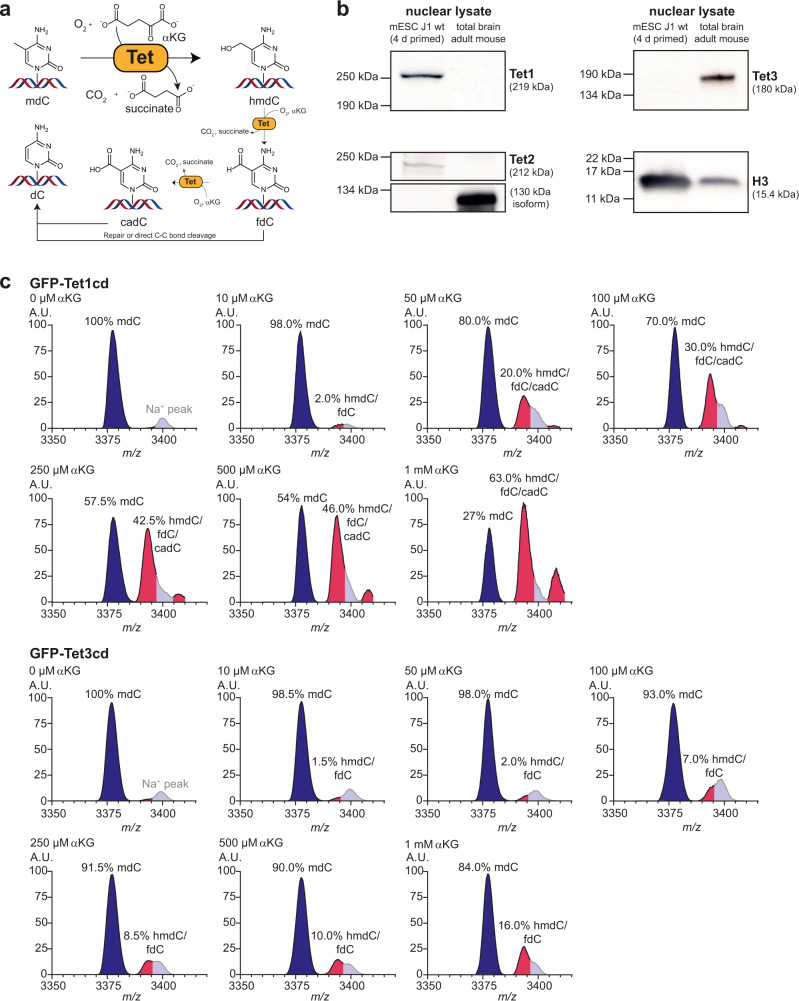
Tet enzyme kinetics. **a** Illustration of αKG and O_2_-dependent mdC-oxidation to hmdC and further on to fdC and cadC by Tet enzymes, with hmdC being the main oxidation product. FdC and cadC can be finally replaced by unmodified dC. **b** Western blots against Tet1, Tet2, and Tet3 in nuclear lysate of mESCs compared to murine brain. **c** MALDI-TOF MS spectra (*n* = 3 independent experiments, spectra show mean) of a single-stranded DNA oligonucleotide containing mdC (blue and Na^+^ peak in gray) that is oxidized to hmdC, fdC, and cadC (magenta) by GFP-Tet1cd or GFP-Tet3cd in the presence of increasing αKG concentrations. A.U. arbitrary units, m/z mass to charge ratio. Source data are provided as a Source Data file.

Genomic DNA (gDNA) in higher vertebrates contains in addition to the four canonical nucleobases 5-methylcytosine (mdC), 5-hydroxymethylcytosine (hmdC), 5-formyl-cytosine (fdC), and 5-carboxy-cytosine (cadC)^1–3^. These cytosine (dC) derivatives give rise to an additional information layer that controls transcriptional activity^4^. The mdC-oxidation products hmdC, fdC, and cadC are generated by ten eleven translocation enzymes (Tet1-3), which are themselves α-ketoglutarate (αKG)- and oxygen-dependent dioxygenases (Fig. 1a). They decarboxylate αKG to generate an active site-bound Fe(IV)-oxo species that ultimately performs the oxidation^5^. Accumulating evidence suggests that mdC and hmdC directly affect transcriptional activity^6^. In contrast, fdC and cadC are considered as nucleobases that are exchanged with dC^7^. This exchange enables active demethylation that allows the system to switch between transcriptional states. While fdC and cadC are only present in trace amounts in differentiated cells, mdC and hmdC are detectable in all tissues^8^. Among all tissues, hmdC levels are by far the highest in brain^9,10^, and in particular found in synapse-related genes of neurons^11^. This correlates with high Tet expression levels in neurons^12^, further suggesting that dynamic oxidation-dependent active DNA demethylation is an essential prerequisite for neuronal plasticity^13,14^. The question of how neuronal nuclei are supplied with the required amounts of αKG is an unsolved question that couples mdC-oxidation chemistry in the genome to metabolism.

αKG is mainly generated in mitochondria as a key intermediate of the tricarboxylic acid (TCA) cycle^15^. The αKG levels outside the mitochondria vary substantially between cell types^16,17^. In differentiating cells this concentration was shown to be about 15 µM and the αKG availability was able to be linked to Tet activity with elevated αKG concentrations providing higher hmdC levels^16,18^. This indicates that αKG supply is indeed a Tet-activity limiting factor^16,18,19^. The principle that co-substrate availability in the nucleus determines the activity of epigenetic enzymes was already established in recent years for other epigenetic marks. Histone acetylation, for example, depends on acetyl-CoA. In neuronal nuclei, acetyl-coA is produced on-site by the acetate-dependent acetyl-CoA synthetase 2, which translocates from the cytosol to the nucleus^20^. In proliferating cells, acetyl-CoA is produced by the pyruvate dehydrogenase complex (PDC), which is shuttled from mitochondria into the nucleus^21^. This moonlighting of mitochondrial and cytosolic protein echoforms into the nucleus is a new principle that establishes the nucleus as a biosynthetically active entity^22^ controlled by metabolic fluctuations that can differ substantially within the cell^23^. Here we show that neuronal Tet3 redirects the mitochondrial enzyme Gdh into the nucleus to establish an intranuclear production of αKG from NAD^+^ and glutamate.

## Results

### Tet3 enzyme kinetics

All three Tet paralogues have functions in a neuronal context^14,24,25^, but only the *Tet3*^−*/−*^ phenotype is neonatal lethal in mice^26,27^. In humans, TET3 deficiencies were further recently linked to intellectual disabilities and growth retardation^28^. In order to compare the global expression levels of all three Tet enzymes in murine brain, a western blot was performed and the Tet expression levels in brain were compared to the levels in mouse embryonic stem cells (mESCs), which are known to have high Tet1, but low Tet3 levels^3^. The data depicted in Fig. 1b confirm that in accordance with literature^12^, Tet3 is compared to Tet1 the dominantly expressed paralogue in brain. We next studied the affinity of Tet3 for αKG; again in comparison to Tet1 for which the enzyme kinetics were already studied in detail^3,29^. We expressed the catalytic domains of Tet1 and Tet3 as N-terminal GFP-fusion constructs (GFP-Tet1cd and GFP-Tet3cd) in HEK293T cells and purified the proteins with anti-GFP nanobody-coated magnetic beads (Supplementary Fig. 1a). For the assay, we adjusted the amounts of added protein based on the GFP fluorescence. The bead-attached proteins were then used to oxidize a single-stranded DNA oligonucleotide substrate containing one central mdC base with increasing amounts of αKG (Fig. 1c). The DNA was subsequently purified and the conversion of mdC to the three oxidation products hmdC, fdC, and cadC was analyzed by matrix-assisted laser desorption ionization time of flight (MALDI-TOF) and quantified on the nucleoside level by UHPLC triple quadrupole (UHPLC-QQQ) mass spectrometry (MS). The MALDI data revealed that GFP-Tet3cd requires an approximately 20-fold higher αKG concentration than GFP-Tet1cd to reach a similar oxidation yield (Fig. 1c). Our UHPLC-QQQ-MS data revealed a Michaelis constant (*K*_m_) of 39 µM αKG for GFP-Tet1cd, which is slightly lower than the previously reported *K*_m_ for Tet1^29^. For GFP-Tet3cd, the measured *K*_m_ was almost twofold higher at 73 µM αKG with a maximal conversion rate (*v*_max_) only 15% compared to GFP-Tet1cd for (Supplementary Fig. 1b). In summary, the data suggest that Tet3, which is the dominantly expressed Tet paralogue in brain, has a particularly low affinity for αKG.

### Gdh produces αKG co-substrate for Tet3 directly in neuronal nuclei

**Fig. 2:**
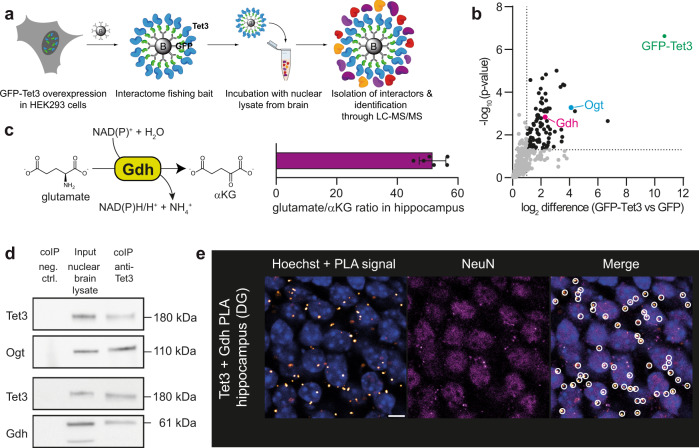
Interaction of Tet3 with Gdh in neurons. **a** Experimental set-up for Tet3-enriched coIP. **b** Volcano plot after Tet3-enriched coIP in nuclear brain lysate of adult mice (*n* = 4 biologically independent sample preparations). Interaction partners were analyzed using label-free quantification after LC–MS/MS. Enriched interaction partners (FDR < 0.05, log_2_ FC > 1) are highlighted in black. **c** Conversion of glutamate to αKG by Gdh and glutamate to αKG ratio determined by NMR-based metabolomics in murine hippocampus (*n* = 6 biologically independent animals, bar shows mean, error bars SD). **d** Western blots detecting Tet3, Ogt, and Gdh after endogenous Tet3-coIP in nuclear brain lysate. **e** PLA signal (white/orange dots) of Tet3 and Gdh in the dentate gyrus (DG) of the murine hippocampus. Nuclei (blue) were stained with Hoechst and NeuN staining indicates neuronal nuclei. Scale bar is 5 µm. Source data are provided as a Source Data file.

In order to search for Tet3 interaction partners that could provide αKG, we next performed an affinity-based proteomics study with nuclear lysate from whole murine brain. Prior to the study, we confirmed that the lysates showed specific enrichment of nuclear proteins (Supplementary Fig. 1c). For the initial co-immunoprecipitation (coIP), we overexpressed murine full-length Tet3 lacking the CXXC domain, which is the prevalent isoform in neuronal tissue^30^, as an N-terminal GFP-fusion construct (GFP-Tet3) in HEK293T cells. The protein was then immobilized again on anti-GFP nanobody-coated beads (Supplementary Fig. 1d, e) and the beads were used as fishing baits for interacting proteins (Fig. 2a). Unfused GFP purified from the same source served as a negative control. By this approach we aimed to overcome the potential problem of antibody specificity, which may occur if two different antibodies—one for the actual coIP and one for the control coIP—are used. The samples were analyzed using label-free quantitative mass spectrometry (LFQ-MS)^31^. The statistical analysis of the data confirmed that the method provided highly reproducible datasets. The obtained data showed a high correlation within the replicates as determined by the Pearson correlation (Supplementary Fig. 2a). Particularly informative was the enrichment of O-linked-β-N-acetylglucosamine-transferase (Ogt) (Fig. 2b and Supplementary Fig. 2b). Because Ogt is a well-known Tet3 interactor^32–34^, this finding was a first validation of the method. A search for other enriched proteins uncovered the mitochondrial enzyme glutamate dehydrogenase (Gdh), which catalyzes the NAD^+^-dependent conversion of glutamate into αKG (Fig. 2c), as a Tet3 interactor (Fig. 2b and Supplementary Fig. 2b). Gdh is a key component of carbohydrate, amino acid, neurotransmitter and oxidative energy metabolism^35,36^ and typically localizes to mitochondria^37^. The discovery of Gdh as a Tet3 interactor in nuclear lysate suggests an additional nuclear localization of Gdh.

Given the very high glutamate concentration in neurons, ranging from 5 to 10 mM^23^, an on-site biosynthesis of αKG from glutamate by nuclear Gdh would establish a direct local co-substrate supply chain for Tet3. Gdh is a bidirectional enzyme that can also consume αKG. For this to occur, however, Gdh requires low glutamate concentrations and especially high levels of ammonium, which are known to be toxic for neurons^38,39^. To address the enzyme directionality, we measured the αKG-to-glutamate ratio by NMR-based metabolomics^40^ in hippocampus. We found that the glutamate levels are least 50 times higher than those for αKG (Fig. 2c). This finding in combination with the low ammonium levels in neurons^39^ suggests that Gdh is operating in neuronal nuclei in the direction of αKG production, which is in line with previous reports about the function of Gdh in rat brain^41^.

To substantiate the suspected interaction of Tet3 with Gdh, we immunoprecipitated endogenous Tet3 from nuclear brain lysate and analyzed the interaction partners by western blotting. The interaction of Tet3 with Ogt served again as a positive control. Importantly, Gdh was successfully pulled down in Tet3-endogenous coIP (Fig. 2d), confirming the result obtained by Tet3-enriched coIP. To further validate the interaction of Tet3 and Gdh in situ, we next performed a proximity ligation assay (PLA), which provides a signal only when the two investigated proteins are within a 40 nm distance^42^. For the study, we used coronal brain slices and antibodies that were validated before the experiment (Supplementary Fig. 3 and ref. ^43^). Indeed, the Tet3/Gdh-PLA provided positive signals in hippocampal neurons (Fig. 2e and Supplementary Fig. 4a), further supporting a close proximity of Tet3 and Gdh in neuronal nuclei.

### Gdh is transported with its MTS into neuronal nuclei

**Fig. 3:**
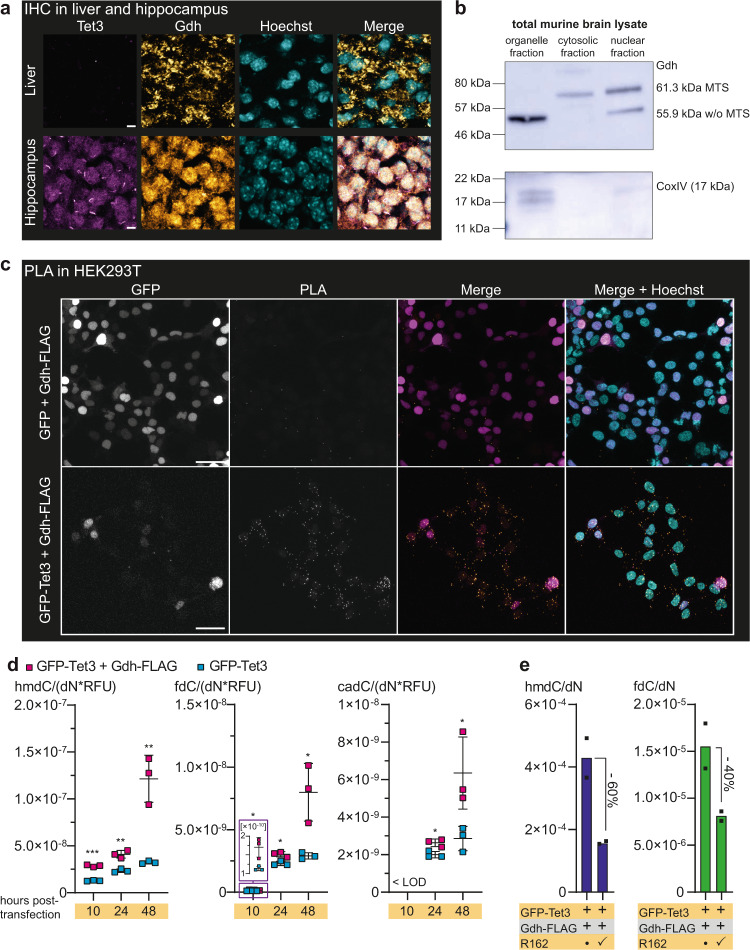
Gdh localization in brain and liver and Tet3 + Gdh interaction after ectopic co-expression in HEK293T cells. **a** Immunofluorescence staining of Tet3 and Gdh in murine hippocampus compared to liver. Scale bar is 5 µm. **b** Western blot to determine Gdh content of different cellular compartments (fractionation from total murine brain) shows different echoforms of Gdh. CoxIV was used as a mitochondrial marker. **c** PLA in HEK293T after ectopic co-expression of GFP-Tet3 and Gdh-FLAG. GFP + Gdh-FLAG co-expressing cells were used as a negative control. In the merged image, GFP signal is shown in magenta, PLA signal in orange and nuclei (cyan) were stained with Hoechst. Scale bar is 50 µm. **d** Levels of hmdC, fdC, and cadC 10 h, 24 h, and 48 h after transfection of HEK293T (*n* = 3 biologically independent samples for each timepoint) with either GFP-Tet3 + Gdh-FLAG expressing plasmids (magenta) or GFP-Tet3 expression plasmid and an empty vector (cyan). Levels of mdC-oxidation products were normalized to the GFP signal of the cells. For each modification, each timepoint was compared individually. Two-sided *t*-test, correction for multiple comparisons using Holm-Sidak method, *p*_adj_ < 0.05 (*), <0.01 (**), <0.001 (***); exact *p* values and statistical details are provided in the Statistics and Reproducibility sub-section within the “Methods” section. Bars show mean, error bars show SD. LOD limit of detection **e** Levels of hmdC and fdC in the absence or presence of 20 µM of Gdh inhibitor R162 24 h after transfection of HEK293T (*n* = 2 biologically independent samples) with GFP-Tet3 and Gdh-FLAG expressing plasmids. **a**, **c** Images show Z-stacks. Source data are provided as a Source Data file.

Gdh is known to be highly expressed in brain, which has high hmdC and Tet3 levels, as well as in liver, which has in contrast very low hmdC and Tet3 levels in a non-fasting state^9,12,41,44^. We investigated if the differences regarding the hmdC levels correlate with a different Gdh distribution. Using immunohistochemistry (IHC), we indeed found an exclusive mitochondrial localization for Gdh in hepatocytes. A specific Gdh signal in the nuclei of hepatocytes could not be detected and we failed to detect Tet3 (Fig. 3a and Supplementary Fig. 4b). In granule neurons of the hippocampal dentate gyrus, however, we detected only very little Gdh in mitochondria. In contrast, Gdh was dominantly present in the nucleus. Tet3 was also detected with the expected nuclear localization (Fig. 3a and Supplementary Fig. 4b). Altogether, these immunofluorescence data revealed that Gdh exists in two echoforms of which one is situated inside mitochondria, while a second form can be transferred to the nucleus in neurons.

Next, we investigated the question of how Gdh can moonlight^22^ into the nucleus. The gene *Glud1* encodes Gdh including an N-terminal 53 amino acids long mitochondrial-targeting sequence (MTS), which is cleaved off after import into the mitochondria^35,37^. When we analyzed neuronal Gdh in different subcellular compartments by western blotting, we found in the cytosolic fraction only the long variant with MTS migrating above the 57 kDa marker band, whereas in the organelle fraction only the short variant without MTS was detected (Fig. 3b). For the nuclear fraction, we detected two Gdh signals, with the dominant signal being at ~61.3 kDa and the weaker signal at ~55.9 kDa corresponding to two Gdh echoforms with and without the MTS, respectively. The weak band below 57 kDa that is detected in the nuclear fraction likely reflects some mitochondrial impurity, which is supported by a weak signal for CoxIV, which is a protein of the mitochondrial matrix. As the MTS is cleaved off after its import into mitochondria, detection of the short version in the organelle fraction was expected. The discovery of the long Gdh echoform in the nucleus suggests that the nuclear Gdh does not pass through the mitochondrion, but rather that it is directly transferred into the nucleus despite its MTS.

### The Gdh-Tet3 proximity increases Tet3 activity

To study how the proximity of Tet3 and Gdh influences Tet3 function, we performed a functional assay in HEK293T cells with ectopically co-expressed murine GFP-Tet3 and C-terminally FLAG-tagged murine Gdh (Gdh-FLAG). We first verified the interaction of GFP-Tet3 and Gdh-FLAG in HEK293T cells, again using a PLA with anti-GFP and anti-FLAG primary antibodies (Fig. 3c). While in the negative control, where unfused GFP and Gdh-FLAG were co-expressed, no PLA signal was detectable, co-expression of GFP-Tet3 and Gdh-FLAG provided clear PLA signals, indicating that GFP-Tet3 and Gdh-FLAG were in close proximity in the nuclei of HEK293T cells. Next, we quantified the levels of hmdC, fdC, and cadC using our reported UHPLC-QQQ-MS method^45^ at 10, 24, and 48 h post transfection in HEK293T cells that were either transfected with plasmids coding for GFP-Tet3 and Gdh-FLAG, or with the plasmid coding for GFP-Tet3 and an empty vector. In this model system, glutamate supply was guaranteed by the addition of 2 mM of l-alanyl-glutamine to the medium. To correct for expression level differences of GFP-Tet3 between the samples, we normalized the hmdC, fdC, and cadC levels against the GFP signal (relative fluorescent unit (RFU)), which we determined using fluorescence-based flow cytometry (Supplementary Fig. 5a). For all modifications at any timepoint, the GFP-Tet3 + Gdh-FLAG co-expressing HEK293T cells showed significantly higher Tet-product levels than the cells expressing GFP-Tet3 alone (Fig. 3d). The largest difference was observed after 48 h, indicating that under limited nutrient and therefore limited αKG availability, additional αKG supply by Gdh enhances the Tet3 activity substantially.

To further investigate the stimulatory effect of Gdh activity on Tet3, we applied the previously published Gdh inhibitor R162 on the GFP-Tet3/Gdh-FLAG expressing cells. R162, which is a purpurin analog, was shown to be a highly specific Gdh inhibitor with an inhibition and a dissociation constant (*K*_i_ and *K*_d_) of around 30 µM and a good cell permeability due to its allyl group^46^. In the presence of R162, the hmdC levels dropped by 60% and the fdC levels dropped by 40% (Fig. 3e). Application of R162 did not decrease Tet3 activity in the cells expressing only GFP-Tet3 (Supplementary Fig. 5b), indicating that the endogenous (human) GDH does not contribute to the αKG supply of ectopically expressed murine Tet3.

**Fig. 4:**
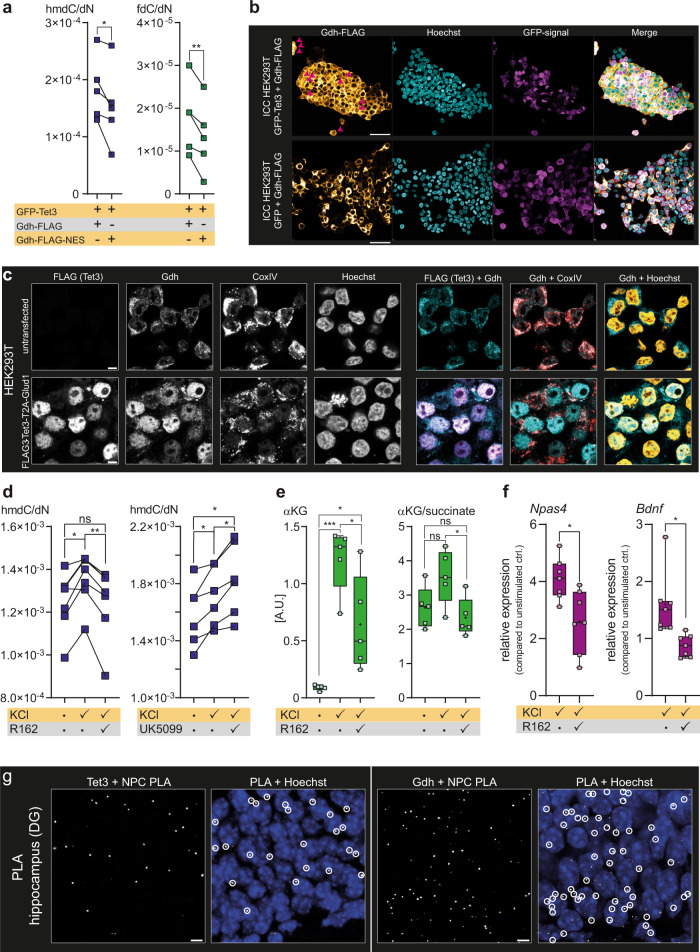
Gdh localization and influence of Gdh on Tet enzymes, gene expression and metabolism during neuronal stimulation. **a** Comparison of hmdC and fdC levels in HEK293T (*n* = 5 biologically independent samples) expressing GFP-Tet3 + Gdh-FLAG or GFP-Tet3 + Gdh-FLAG-NES for 24 h. A two-sided paired *t*-test was performed. **b** ICC in HEK239T showing FLAG and GFP signal after ectopic co-expression of GFP-Tet3 + Gdh-FLAG or GFP + Gdh-FLAG from different promoters. Scale bar is 50 µm. **c** ICC in HEK293T showing FLAG, Gdh, and CoxIV after expressing FLAG_3_-Tet3 and Gdh from a bicistronic vector compared to untransfected cells. Merged images: FLAG (magenta), Gdh (cyan), CoxIV (red) and Hoechst (yellow). Scale bar is 5 µm. **d** Hippocampal hmdC levels upon neuronal stimulation with 25 mM KCl for 6 h with and without 20 µM Gdh inhibitor R162 (*n* = 7 biologically independent animals) or 40 µM PDC inhibitor UK5099 (*n* = 6 biologically independent animals). Connected dots represent matched hmdC values from one individual mouse. RM one-way ANOVA combined with Tukey’s multiple comparisons test was performed. **e** αKG level and αKG/succinate ratio in hippocampus (*n* = 5 biologically independent animals) after depolarization with and without R162. Ordinary one-way ANOVA combined with Tukey’s multiple comparisons test was performed. A.U. arbitrary units. **f** Relative gene expression levels of Npas4 and Bdnf (*n* = 7 biologically independent animals) in hippocampus after depolarization with and without R162 normalized to expression levels in unstimulated control. An unpaired two-sided t-test with Welch’s correction was performed. **g** PLA of Tet3 **+** NPC and Gdh + NPC in the hippocampus. Images show Z-stacks. Scale bar is 5 µm. **a**, **d**–**f** *p* (a, f) or *p*_(adj)_ (**d**, **e**) <0.05 (*), <0.01 (**), <0.001 (***); exact *p* values and statistical details are provided in the Statistics and Reproducibility sub-section within the “Methods” section. **e**, **f** Boxes display interquartile range, median is shown as center line, mean as + , whiskers represent the minimum and the maximum. Source data are provided as a Source Data file.

In order to check how the proximity of Gdh and Tet3 influences Tet3 activity, we repeated the experiment with a Gdh-FLAG construct additionally containing a nuclear export sequence (Gdh-FLAG-NES), to prevent nuclear localization of Gdh (Supplementary Fig. 5c). We co-expressed Gdh-FLAG-NES with GFP-Tet3 and measured the hmdC and fdC levels compared to the GFP-Tet3/Gdh-FLAG expressing HEK293T cells. Twenty-four hours post transfection, the hmdC and fdC levels were significantly lower in the GFP-Tet3 + Gdh-FLAG-NES expressing cells (Fig. 4a). In addition, we were not able to mimic the effect of Gdh on Tet3 by the addition of 4 mM of cell-permeable dimethyl-αKG (DM-αKG) (Supplementary Fig. 5d), suggesting that the direct supply of Tet3 with αKG by Gdh in proximity is indeed enhancing Tet3 activity.

To clarify whether Gdh transport into the nucleus depends on Tet3, we next investigated the localization of Gdh in GFP-Tet3 + Gdh-FLAG and GFP + Gdh-FLAG expressing HEK293T cells using immunocytochemistry (ICC). Interestingly, when Gdh-FLAG was co-expressed with GFP-Tet3, Gdh-FLAG localization was ~40% nuclear and 60% mitochondrial (Fig. 4b and Supplementary Fig. 6a). GFP-Tet3 showed only the expected nuclear localization. In contrast, nuclear localization of Gdh-FLAG was abolished when it was co-expressed with GFP alone (Fig. 4b), indicating that the Gdh import into the nucleus is Tet3-dependent. We noted, however, that the expression of Tet3 and Gdh from two different promoters provided much higher Gdh levels than Tet3, which compromised interpretation of the data. Therefore, we repeated the experiment with a bicistronic vector including a T2A sequence that allowed for equimolar expression of FLAG_3_-Tet3 and Gdh from the same promoter. Previous studies confirmed that ribosome skipping at the T2A is very efficient, so that only low amounts of fusion proteins are formed, and that this expression system does not change the localization of the expressed proteins^47,48^. Using this expression system, we first investigated the possible formation of Tet3-Gdh fusion proteins and found none by western blotting, whereas a forced Tet3-Gdh fusion protein using the bicistronic vector with a mutated T2A sequence (T2A^ΔGP^), which prevents the ribosome skipping event, showed a clear band at the expected size (Supplementary Fig. 6b). The following ICC studies revealed that in the presence of Tet3, Gdh was efficiently transported to the nucleus and only minor amounts of Gdh were detected in mitochondria. By comparison, the endogenous human GDH was only located in the mitochondria (Fig. 4c), which might explain why the application of R162 did not change the Tet3 activity in the just GFP-Tet3 expressing cells. The forced Tet3-Gdh fusion protein did not locate to the nucleus but remained in the cytosol (Supplementary Fig. 6c). Altogether, the data support our model that the nuclear localization of Gdh requires the presence of Tet3 and suggests that Tet3 itself, or a complex of which Tet3 is part of, mediates transfer of Gdh into the nucleus.

### Neuronal depolarization leads to Gdh-dependent stimulation of Tet enzymes

To transfer these findings from the HEK293T model system into a more physiological context, we exposed acute mouse hippocampal slices to depolarizing conditions (25 mM KCl), which simulate stimulation and induce neural activity^49^. After 6 h, we quantified the hmdC levels and compared them to the hmdC levels of hippocampal slices that were kept in parallel in a physiological buffer solution. Constant neuronal depolarization led to significantly increased global hmdC levels. In the presence of the Gdh inhibitor R162, however, this hmdC increase could be fully suppressed (Fig. 4d). Although off-target effects of the pharmacological small-molecule inhibitor R162 could not be fully excluded, previous publications gave no such indication^46^. Importantly, application of R162 allowed us to influence Gdh activity on the protein level directly and immediately. In contrast, siRNA-based approaches to knock-down Gdh expression by initiating Gdh-mRNA degradation do not affect Gdh activity directly and require transfection of siRNA, which might have comprised the quality of the sensible acute hippocampal slices.

As a reversed approach, we applied UK5099, which acts as an inhibitor of the mitochondrial pyruvate carrier and as a stimulator of Gdh^50^. In this experimental set-up, a further increase in the hmdC levels after stimulation was observed (Fig. 3d). These data suggest that Gdh is indeed involved in Tet-dependent hmdC generation upon neuronal activation.

Quantification of αKG in the hippocampal slices before and after depolarization supported this interpretation. We detected a strong increase of the αKG concentration and an increase of the αKG/succinate ratio under depolarizing conditions. In the presence of the Gdh inhibitor R162, however, the αKG amount dropped significantly, while the αKG/succinate levels were back at the basal level (Fig. 4e). These data suggest that a substantial amount of the αKG increase during neural activity is caused by the action of Gdh.

We wanted to explore whether the metabolic stimulation of Tet-activity affects the expression levels of important neuronal genes. Neuronal stimulation is known to affect the DNA methylation status of several genes that modulate the response of the neuron toward present and future stimuli^13,14^. Among those genes, the neuronal PAS-domain containing protein 4 (Npas4) and the brain-derived neurotrophic factor (Bdnf) play a pivotal role in synaptic homeostasis, learning and memory formation^51,52^. We used RT-qPCR to quantify the expression of *Npas4* and *Bdnf* and observed for both genes reduced transcription during neuronal stimulation upon addition of R162 (Fig. 4f).

It is known that spatial organization of the nucleus has a direct impact on gene expression and that localization of a gene at the nuclear laminar is associated with gene silencing, whereas proximity to the nuclear pore complex (NPC) is linked to active gene expression^53^. Therefore, the gene-activating interaction of Tet3 and Gdh would be expected to proceed at the NPC. We noticed that a high number of Tet3 + Gdh-PLA signals in the hippocampus derived from the nuclear periphery (Fig. 2e). To test for the hypothesis that the interaction between Tet3 and Gdh happens at the NPC, we investigated the individual interactions of Tet3 and Gdh with the NPC by PLA. Indeed, in both cases, we obtained PLA signals supporting that the Tet3/Gdh complex is localized close to the NPC (Fig. 4g). In addition, proteomic analysis of a Tet3-coIP in nuclear brain lysate showed enrichment of several RNA-processing proteins like Fus (Supplementary Fig. 2b), which is a DNA/RNA-binding protein that is important for pre-mRNA binding and transport and has distinct functions in neuronal homeostasis^54^. However, how the interaction of Tet3 and Gdh is organized within the nucleus and how this spatial organization is specifically linked to gene expression, needs to be addressed in future studies.

Altogether, our data support the idea that the interplay between nuclear Gdh for the NAD^+^-dependent manufacturing of αKG and Tet3 is a hinge that allows metabolism to influence active demethylation processes in neurons. The mechanisms how the interaction of Tet3 and Gdh is regulated and whether Tet3 and Gdh interact indirectly or directly, potentially as part of a larger complex requires further investigation.

## Discussion

Gdh is responsible for the NAD^+^-dependent deamination of glutamate to αKG. Although it is known to be a mitochondrial enzyme and is expressed with an MTS, Gdh is reported to have an additional nuclear localization in neural contexts^35,37,55^. Our data confirm that neuronal Gdh has indeed a split spatial distribution with a substantial fraction of the protein being redirected to the nucleus, probably before entering the mitochondria. In neurons, glutamate is one of the most abundant metabolites making it in this respect an ideal starting material for the generation of large amounts of αKG. Our data suggest that one of the functions of Gdh in the nucleus is to supply Tet3 with αKG, thereby regulating Tet3-activity. Given that αKG is involved in many epigenetic processes and energy metabolism in general^15^, elevating the effective αKG molarity solely in the presence of Tet3 is an elegant way to avoid globally changing αKG levels, which could produce various side effects. This puts Gdh in line with other mitochondrial proteins, such as the PDC or enzymes of the TCA cycle that can be also translocated to the nucleus for controlled intranuclear metabolite biosynthesis and consequently metabolic regulation of gene expression by changing histone modifications^21,56^. Our data expand this coupling between metabolism and epigenetics to the Tet-dependent oxidation of mdC to hmdC and likely further on to fdC and cadC. Since Gdh requires NAD^+^ for the conversion of glutamate into αKG, these oxidation reactions are seemingly associated with the NAD^+^ levels in the nucleus. NAD^+^ is an essential small-molecule cofactor that is biosynthesized in a compartmentalized manner by different nicotinamide mononucleotide adenylyl transferases (NMNATs) in the nucleus (NMNAT-1), in the cytoplasm (NMNAT-2) and in mitochondria (NMNAT-3)^57^. It was recently shown that the nuclear NAD^+^ levels fluctuate depending on the metabolic state of the cell and it was suggested that the compartmentalized NAD^+^ biosynthesis plays a key role in controlling transcriptional processes^58^. Although we are far from understanding how a shift in energy and NAD^+^ metabolism leads to the controlled activation of specific genes, our data suggest that nuclear localized Gdh for the NAD^+^-dependent on-site biosynthesis of αKG to steer Tet3 activity, can be an important contributor and regulator.

## Methods

All procedures concerning animals conform to the German animal protection laws and were approved by the local authority (Regierung von Oberbayern).

### Antibodies

CoxIV antibody (Cell Signaling Technology brain-derived neurotrophic fact4850, clone 3E11, rabbit monoclonal IgG): western blotting (1:1000), ICC (1:500)

Cytochrome C antibody (Santa Cruz Biotechnology sc-13560, clone 7H8, mouse monoclonal IgG): western blotting (1:1000)

FLAG antibody (Sigma-Aldrich F1804, clone M2, mouse monoclonal IgG): western blotting (1:2000), ICC (1:500)

FLAG antibody (Sigma-Aldrich F2555, clone SIG1-25, rabbit monoclonal IgG): PLA (1:250)

Gdh antibody (Invitrogen PA5-19267, goat polyclonal IgG, Lot #74422112, P1): western blotting (1:1000 – 1:2000), ICC (1:100 – 1:200), IHC (1:100)

Validation:

- Western blotting: signal at 56 kDa for haploid mESC, no signal for haploid mESC^Glud1 -/^ (Supplementary Fig. 3d)
- ICC: strong signal for haploid mESC, no signal for haploid mESC^*Glud1* -/^ (Supplementary Fig. 3d)

GFP antibody (Cell Signaling Technology 2955, clone 4B10, mouse monoclonal): western blotting (1:1000), PLA (1:200)

Histone H3 antibody (Cell Signaling Technology 4499 S, clone D1H2, rabbit monoclonal IgG): western blotting 1:1000

NeuN antibody (EMD Millipore MAB377X, clone A60, Alexa488 conjugated, mouse monoclonal IgG): IHC (1:100)

NPC antibody (Sigma-Aldrich N8786, clone 414, mouse monoclonal IgG): IHC (1:250)

Ogt antibody (Invitrogen PA5-22071, rabbit polyclonal IgG, Lot #RH2258725): western blotting (1:1000)

Tet1 antibody (Active Motif 61741, clone 5D6, rat monoclonal IgG): western blotting (1:1000)

Validation:

- Western blotting: one strong signal above 200 kDa for mESC, but not for mESC TET triple knockout (TKO) cells (Supplementary Fig. 7)

Tet2 antibody (ptglab 21207-1-AP, rabbit polyclonal IgG): western blotting (1:1000)

Validation:

- Western blotting: a strong signal at the expected 212 kDa for mESC and at 130 kDa for murine brain, but not for mESC TET TKO cells (Supplementary Fig. 7)

Tet3 antibody (Abiocode N1 R1092-1, rabbit polyclonal IgG, Lot #7063 and #9013):

western blotting (1:1000), coIP (2 µg), ICC (1:500), IHC (1:500)

Validation:

- Western blotting: one strong signal at ~180 kDa for nuclear brain lysate, but not for mESC TET TKO cells (Supplementary Fig. 3a)
- ICC: total signal overlap with GFP in GFP-Tet3 transfected HEK293T, but not with GFP in GFP only transfected HEK293T (Supplementary Fig. 3b)
- ICC: no signal in mESC TET TKO (Supplementary Fig. 3c)
- IP: validation for IP has already been reported^43^

β-Tubulin antibody (Cell Signaling Technology 2128, clone 9F3, rabbit monoclonal IgG): western blotting (1:1000)

### Secondary antibodies

Anti-goat IgG (Sigma-Aldrich G4018, rabbit polyclonal): IP (1 µg for MS analysis, 2 µg for western blot analysis)

Cy2-anti-goat IgG (Jackson ImmunoResearch 705-225-147): IHC (1:200)

Cy3-anti-goat IgG (Jackson ImmunoResearch 805-165-180): IHC (1:400)

HRP-conjugated anti-goat IgG (Sigma-Aldrich A5420): western blotting (1:5000)

Alexa488-anti-mouse IgG (Cell Signaling Technologies 4408): IHC (1:800)

Alexa555-anti-mouse IgG (Cell Signaling Technologies 4409): IHC (1:800)

HRP-conjugated anti-mouse IgG (Sigma-Aldrich AP130P): western blotting (1:5000)

Cy3-anti-mouse IgG (Jackson ImmunoResearch 715-165-150): IHC (1:400)

Alexa488-anti-rabbit IgG (Cell Signaling Technologies 4412): IHC (1:800)

Alexa555-anti-rabbit IgG (Cell Signaling Technologies 4413): IHC (1:800)

Cy3-anti-rabbit IgG (Jackson ImmunoResearch 711-165-152): IHC (1:400)

HRP-conjugated anti-rabbit IgG (Sigma-Aldrich A0545): western blotting (1:5000)

### Expression plasmids

GFP-Tet1cd (plasmid with ampicillin resistance, CAG promoter^59^):

Expressed protein (N-terminus) eGFP—TEV cleavage site—Tet1cd (C-terminus); Tet1cd is the shortened version of murine Tet1 (NCBI XP_006513930.1) starting from amino acid 1367 of the original sequence, TEV cleavage site ENLYFQ|G

GFP-Tet3cd (plasmid with ampicillin resistance, CAG promoter):

Expressed protein (N-terminus) eGFP—TEV cleavage site—Tet3cd (C-terminus); Tet3cd is the shortened version of murine Tet3 (NCBI NP_001334242.1) starting from amino acid 696 of the original sequence

GFP-Tet3 (plasmid with ampicillin resistance, CAG promoter^59^):

Expressed protein (N-terminus) eGFP—TEV cleavage site—Tet3; murine Tet3 (NP_898961.2) starting from amino acid 1 of the original sequence

Glud1-FLAG (plasmid with ampicillin resistance, CMV promoter):

Expressed protein (N-terminus) Gdh-FLAG (C-terminus); murine Glud1 (UniProtKB – P26443) starting from amino acid 1 of the original sequence, FLAG DYKDDDDK

Glud1-FLAG-NES (plasmid with ampicillin resistance, CMV promoter):

Expressed protein (N-terminus) Gdh-FLAG-NES (C-terminus); NES GS**LALKLAGLDI**^60^

FLAG_3_-Tet3-T2A-Glud1 (plasmid with ampicillin resistance, promoter TRE):

Expressed proteins (N-terminus) Strep-FLAG_3_ - TEV cleavage site - Tet3 (C-terminus) and Gdh

FLAG_3_-Tet3-T2A^ΔGP^-Glud1 (plasmid with ampicillin resistance, promoter TRE):

Expressed protein (N-terminus) Strep-FLAG_3_ - TEV cleavage site -Tet3-Gdh (C-terminus)

### Chemical structures

Chemical structures were drawn using ChemDraw Professional 16.0.

### Cell culture HEK293T cells

HEK293T cells (ATCC) were cultivated at 37 °C in water saturated, CO_2_-enriched (5%) atmosphere. DMEM (Sigma-Aldrich D6546) or RPMI 1640 (Sigma-Aldrich R0883), containing 10% (v/v) fetal bovine serum (Invitrogen 10500-064), 1% (v/v) l-alanyl-l-glutamine (Sigma-Aldrich G8541), and 1% (v/v) penicillin–streptomycin (Sigma-Aldrich P0781), were used as growing medium. When reaching a confluence of 70–80%, the cells were routinely passaged. Cells were tested at least once in 2 months for Mycoplasma contamination using Mycoplasma Detection Kit (JenaBioscience PP-401L).

### Transfection of HEK293T cells

#### Transfection of HEK293T cells for high protein expression

The transfection was performed in four p150 petri dishes (Sarstedt 83.3903.300). Five to six million cells per p150 were seeded in 25 mL of medium. After seeding, the cells were incubated for 24 h to reach a confluence of 40–80%. Ten micrograms of expression plasmid DNA and 30 µL of the transfection reagent jetPRIME (Polyplus Transfection VWR 114-15) were used as described by the manufacturer. Four hours and 28 h after transfection the medium was changed, and sodium butyrate (final conc. 4 mM) was added. Forty-eight hours after transfection, the cells were harvested by trypsinization and immediately used for protein extract preparation.

#### Transfection of HEK293T cells for ICC

The transfection was performed in 15 µ—slide eight-well plates (ibidi 80826). A total of 2–3 × 10^4^ cells were seeded per well in 200 µL of medium. Twenty-four hours after seeding, the cells were transfected using 150 ng of DNA per expression plasmid, 0.5 µL of jetPRIME, and 15 µL of jetPRIME buffer. Twenty-four hours after transfection, the cells were washed once with PBS supplemented with MgCl_2_ and CaCl_2_ (PBS^+^, Dulbecco’s phosphate buffered saline, Sigma-Aldrich D8662) and immunofluorescence staining was performed.

In case of the bicistronic vector construct, cells were transfected with 200 ng plasmid DNA. 4 h after transfection, expression was induced by doxycycline (1 µg/mL final concentration) and 24 h after induction, cells were washed once with PBS^+^ and ICC was performed.

#### Transfection of HEK293T cells for UHPLC-QQQ-MS

The transfection was performed in six-well plates (Sarstedt 83.3920.300). A total of 2.5 × 10^5^ cells were seeded per well in 3 mL of RPMI medium. Twenty-four hours after seeding, the cells were transfected using 1.5 µg of DNA per expression plasmid (combination of GFP-Tet3 and one of the *Glud1* expression plasmids), 4 µL of jetPRIME, and 150 µL of jetPRIME buffer. In case of GFP-Tet3 only expressing cells, 1.5 µg of pESG-iba45 were co-transfected to keep the amount of transfected DNA constant. Six hours after transfection, the medium was changed and 10, 24, and 48 h after transfection, the cells were harvested and washed once with 1 mL of ice-cold PBS. Hundred microliters were used for GFP signal quantification of living cells using FACS (BD LSRFortessa; FSC 130 V, SSC 300 V, GFP 370 V log, 10,000 events per measurement), the rest was lysed and gDNA was isolated and analyzed by UHPLC-QQQ-MS as described previously using the Agilent 6400 Series Triple Quadrupole LC/MS System with the associated MassHunter Workstation Acquisition software and MassHunter Quantitative Analysis software^45^. The hmdC/dN values were subsequently divided by the mean GFP signal to obtain hmdC/(dN*RFU).

When cells were fed with 4 mM DM-αKG, DM-αKG was added with the medium change 6 h after transfection.

### Protein extract preparation

#### Protein extract preparation from transfected HEK293T cells

The harvested HEK293T cells and the resulting lysate were kept on ice during the preparation at all time. Per eight million cells, 1 mL of RIPA buffer (10 mM Tris (pH = 7.5), 150 mM NaCl, 0.5 mM EDTA, 0.1% (w/v) SDS, 1 % (v/v) Triton X-100, 1% (w/v) deoxycholate), supplemented with 2.5 mM MgCl_2_, 100 U/mL benzonase (Merck Millipore 70746-3) and 1 × protease inhibitor cocktail (PIC, Roche 05056489001) on the day of preparation was used for the lysis. Cells were resuspended in RIPA buffer and lysed for 1 h at 4 °C on a tube rotator. Afterward, the lysate was centrifuged (10,000 × *g*, 15 min, 4 °C) and the supernatant containing the proteins was transferred into a new tube. To enrich GFP-tagged proteins, the lysate was immediately incubated with GFP Nano-Traps either on agarose beads (Chromotek gta-20, for in vitro activity assay) or on magnetic agarose beads (Chromotek gtma-20).

#### Protein extract preparations from murine brain

Protein extracts from whole murine brain (Mus musculus, C57-BL6/J wild type, both genders, 110 days old; Charles River, Sulzfeld, Germany), including separation into the organelle, the cytosolic, and the nuclear fraction, were prepared according to a previously published protocol^61^. The nuclear extract was then treated with 25 U/mL benzonase for 30 min on ice and subsequently centrifuged (21,000 × *g*, 15 min, 4 °C). The supernatant containing the nuclear lysate was transferred to a new tube. A Bradford protein assay (Bio-Rad 5000006) was performed according to the manufacturer´s instructions to determine the protein concentration. To check whether the nuclear fraction showed specific enrichment for nuclear proteins in comparison to the combined organelle/cytosolic fraction and was not heavily contaminated with proteins from other compartments, a western blot against histone H3 (nuclear) and cytochrome c (mitochondrial) was performed. The western blot confirmed the specific enrichment of nuclear proteins in the nuclear fraction (Supplementary Fig. 1c).

#### Protein extracts from mESC and HEK293T

Protein extracts from mESC J1 wt, mESC TET TKO (primed for 96 h according to a previously published protocol^62^), and HEK293T were prepared as previously described^63^.

### In vitro activity assay

For the in vitro activity assay on GFP Nano-Trap on agarose beads, GFP-Tet1cd or GFP-Tet3cd bound to the trap was used. The proteins were ectopically expressed in HEK293T (per in vitro assay 1 × P150 culture dishes for GFP-Tet1cd, 3 × P150 culture dishes for GFP-Tet3cd) and the resulting protein extracts were incubated for 1 h at 4 °C on a tube rotator with 140 µL of GFP Nano-Traps on agarose beads per extract. Afterwards, the beads were washed twice with coIP wash buffer 1 (10 mM HEPES pH = 7.5, 150 mM NaCl, 0.5 mM EDTA), twice with coIP wash buffer 2 (10 mM HEPES pH = 7.5, 1 M NaCl, 0.5 mM EDTA) and twice with coIP wash buffer 1. The fluorescence of the GFP-tagged proteins bound to the GFP Nano-Trap was checked on a Tecan Plate Reader (Tecan GENios Pro, fluorescence intensity excitation 400 nm, emission 535 nm) and per reaction, the amount of Nano-Trap was adjusted that the fluorescence signal was 25,000 per reaction. Per assay, seven reactions in TET reaction buffer (50 mM HEPES pH = 7.5, 100 mM NaCl, 2 mM Vitamin C, 1.2 mM ATP, 2.5 mM DTT, 0.1 mM Fe^(II)^(NH_4_)_2_(SO_4_)_2_ 6 × H_2_O) with different concentrations of αKG (0 µM; 10 µM; 50 µM; 100 µM; 250 µM; 500 µM; 1000 µM) and 4 µM of DNA oligonucleotide (5′-TTTTG[mdC]GGTTG-3′) were set up in 50 µL reaction volume/sample. The samples were incubated at 35 °C for 4 h under shaking. For MALDI-TOF measurements, 1 µL of the reaction supernatant was desalted on a 0.025 µm ø VSWP filter membrane against ddH_2_O for at least one hour, co-crystallized in a 3-hydroxypicolinic acid matrix (HPA) and mass spectra were recorded on a Bruker Autoflex II in a *m*/*z* range of 1500–6000. Spectra were normalized to the recorded maximum intensity. For each condition (GFP-Tet1cd or GFP-Tet3cd, defined αKG concentration) three independent experiments were set up and MALDI-TOF spectra recorded. Afterward, the mean was calculated. The area under the curve was calculated using GraphPad Prism (version 8.0.0 or higher) for mdC in the *m*/*z* range 3373.5–3389.0 and 3397.5–3404.5 (Na^+^ peak), hmdC and fdC in the *m*/*z* range 3389.0–3397.5 and cadC in the *m*/*z* range 3404.5–3410.

To determine the αKG concentration where half-maximal conversion rate is achieved, DNA oligonucleotides were purified after the assay using the Oligo Clean & Concentrator (Zymo Research D4061). Fifty picomoles of the DNA oligonucleotide were digested and analyzed by UHPLC-QQQ-MS according to a previously published protocol^45^. As isotopologues D_3_-mdC, D_2_-^15^N_2_-hmdC, ^15^N_2_-fdC, ^15^N_2_-cadC (10 pmol each), and ^13^C_10_-^15^N_2_-dT (120 pmol) were spiked in per 50 µL sample. The absolute amounts [pmol] of hmdC, fdC, cadC were calculated and normalized to the absolute amount of dT. As for the conversion to hmdC one molecule of αKG is needed, for the conversion to fdC two molecules and for the conversion to cadC three molecules, the conversion rate was calculated as (n(hmdC)+2*n(fdC)+3*n(cadC))/n(dT).

### Tet3-enriched coIP

Twenty microliters of magnetic anti-GFP beads (Chromotek gtma-20) were washed three times with GFP wash buffer (10 mM Tris pH 7.5, 150 mM NaCl, 0.5 mM EDTA) and then incubated for 15 min on ice with nuclear extract of GFP-Tet3 overexpressing HEK293T cells. To ensure the saturation of the beads with the GFP-fusion construct, different amounts of lysate were tested and monitored using a Tecan Reader. The GFP-Tet3 loaded beads were then washed twice with coIP wash buffer 1, twice with coIP wash buffer 2 and twice with lysis buffer C (20 mM HEPES pH = 7.5, 420 mM NaCl, 2 mM MgCl_2_, 0.2 mM EDTA, 20% (v/v) glycerol). The GFP-Tet3 beads were subsequently incubated with 200 µg of nuclear brain extract for 15 min on ice. Following, they were washed twice with GFP wash buffer (10 mM Tris pH = 7.5, 150 mM NaCl, 0.5 mM EDTA). To elute the bound proteins, 50 µl of 200 mM glycine pH 2.5 were added and the solution was vortexed for 30 s. To gain more yield, the elution step was repeated. For the negative control, the same procedure was followed using GFP instead of GFP-Tet3.

### Tet3-endogenous coIP

CoIP of endogenous Tet3 was performed using nuclear brain extract. Five hundred micrograms of nuclear brain extract, 2 µg of antibody, and 20 µL of Dynabeads Protein G (Thermo Fisher 10003D) were used per replicate. Anti-goat IgG was used for the negative control, which was performed with the same amounts.

The nuclear brain extract was incubated with the antibody for 1 h at 4 °C on a tube rotator and the Dynabeads Protein G were washed three times with GFP wash buffer. Afterward, the Dynabeads were added to the lysate, PBS was added to a final volume of 500 µL and the suspension was incubated for 1 h at 4 °C on a tube rotator. After incubation, the beads were washed three times 10 min with coIP wash buffer 1. Last, proteins were eluted with 50 µL of SDS loading buffer (50 mM Tris pH 6.8, 100 mM DTT, 2% (w/v) SDS, 10% (v/v) glycerol, 0.1% (w/v) bromophenol blue) for 10 min at 70 °C.

### LC–MS/MS analysis

Samples for the mass spectrometer were reduced by the addition of 100 mM TCEP and subsequent incubation for 1 h at 60 °C on a shaker at 650 rpm. They were then alkylated by adding 200 mM iodoacetamide and incubating for 30 min at room temperature in the dark. Following, the samples were digested with 0.5 µg trypsin (Promega V5113) at 37 °C for 16 h. Afterward, they were incubated for 5 min at 100 °C, and subsequently 1 mM phenylmethylsulphonyl fluoride was added. StageTips were utilized to purify the samples for MS^64^.

The samples were analyzed with an UltiMate 3000 nano liquid chromatography system (Dionex, Fisher Scientific) attached to an LTQ-Orbitrap XL (Thermo Fisher Scientific). They were desalted and concentrated on a µ-precolumn cartridge (PepMap100, C18, 5 µM, 100 Å, size 300 µm i.d. × 5 mm) and further processed on a custom-made analytical column (ReproSil-Pur, C18, 3 µM, 120 Å, packed into a 75 µm i.d. × 150 mm and 8 µm picotip emitter).

The samples were processed via a 127 min multi-step analytical separation at a flow rate of 300 nL/min. The gradient with percentages of solvent B was programmed as follows:

1% for 1 min; 1–4% over 1 min; 4–24% over 100 min; 24–60% over 8 min; 60–85% over 2 min; 85% for 5 min; 85–1% over 2 min; 1% for 8 min.

Mass spectrometric analysis was done with a full mass scan in the mass range between *m*/*z* 300 and 1700 at a resolution of 60,000. Following this survey scan, five scans were performed using the ion trap mass analyzer at a normal resolution setting and wideband CID fragmentation with a normalized collision energy of 35. Signals with an unrecognized charge state or a charge state of 1 were not picked for fragmentation. To avoid supersampling of the peptides, an exclusion list was implemented with the following settings: after 2 measurements in 30 s, the peptide was excluded for 90 s.

### LFQ data processing

The MaxQuant^65^ software (version 1.5.0.25) was used for LFQ. Quantification was performed with four biological replicates for Tet3-enriched coIP. GFP alone (four biological replicates) served here as control. The Andromeda search engine was used in combination with Uniprot databases (Mus musculus). A maximum of two missed cleavage sites was allowed. The main search peptide tolerance was set to 4.5 ppm. Carbamidomethyl (C) was set as static modification. Variable modifications were Acetyl (Protein N-term) and Oxidation (M). The LFQ algorithm was applied with default settings. The option “match between runs” was also used. The MS proteomics data have been deposited to the ProteomeXchange Consortium via the PRIDE^66^ partner repository with the dataset identifier PXD004518.

LFQ data were analyzed with the Perseus software (version 1.5.0.9). The LFQ intensities were log transformed and only proteins identified in at least three samples were retained. As one of the GFP control quadruplicates contained only 64 proteins instead of >400, this replicate was removed from the dataset. Gene ontology analyses were performed with the Database for Annotation, Visualization, and Integrated Discovery (DAVID Bioinformatics Resources 6.7).

### Western blotting

Samples were loaded on a 4–15% precast polyacrylamide gel (Bio-Rad) and MagicMark XP Standard (Thermo Fisher LC5603) and Blue Prestained Protein Standard, Broad Range (11–190 kDa) (New England Biolabs P7706S) or Color-coded Prestained Protein Marker, Broad Range (11–250 kDa) (New England Biolabs 14208) were used as protein standards. The gel was run at constant 150 V for 60 min in SDS running buffer (25 mM Tris, 192 mM glycine, 0.1% (w/v) SDS). For blotting, we used a PVDF blotting membrane (GE Healthcare Amersham Hybond P0.45 PVDF membrane 10600023) and pre-cooled Towbin blotting buffer (25 mM Tris, 192 mM glycine, 20% (v/v) methanol, 0.038% (w/v) SDS). The membrane was activated for 1 min in methanol, washed with water, and equilibrated for additional 1 min in Towbin blotting buffer; the Whatman gel blotting papers (Sigma-Aldrich WHA 10426981) were equilibrated for 15 min in Towbin buffer and the precast gel was equilibrated for 5 min in Towbin buffer after the run. Western blotting (tank (wet) electro transfer) was performed at 4 °C for 10 h at constant 35 V. After blotting, the PVDF membrane was blocked for 1 h at room temperature using 5% (w/v) milk powder in TBS-T (20 mM Tris pH = 7.5, 150 mM NaCl, 0.1% (v/v) Tween-20). The primary antibodies were diluted in 5 mL of 5% (w/v) milk powder in TBS-T. The blocking suspension was discarded, and the diluted primary antibodies were added for 12 h at 4 °C and shaking. After incubation, the primary antibodies were discarded, and the membrane was washed three times 10 min with TBS-T. HRP-conjugated secondary antibodies were diluted in 5% (w/v) milk powder in TBS-T and added for 1 h at room temperature under shaking. In case of Tet3-endogenous coIP samples, TidyBlot HRP-conjugated Western blot detection reagent (Bio-Rad STAR209P) was used instead of HRP-conjugated secondary antibodies if the primary antibody against the potential interactor was produced in rabbit to avoid detection of the antibody fragments from the coIP. Afterward, the membrane was washed two times with TBS-T and one time with TBS (TBS-T without Tween-20) before SuperSignal West Pico Chemiluminescent Substrate (Thermo Scientific 34077) was used for imaging. Western blots were imaged using Amersham Imager 680 (auto exposure mode).

### IHC

All steps were performed in a humidity chamber and at room temperature when not otherwise specified. Twelve micrometers thick coronar cryo-sections of snap-frozen adult mouse brain and liver were incubated (if applicable) with MitoTracker Deep Red FM (Invitrogen M22426) before fixation. Cryo-sections were fixed on slides using 4% paraformaldehyde (4% PFA, Thermo Scientific 28908) in 0.1 M phosphate-buffered solution, pH 7.4 (0.1 M PB). After three times washing with 0.1 M PB, the slices were permeabilized and blocked for 30 min using 0.3% (v/v) Triton X-100 and 5% (v/v) blocking reagent CB (Chemiblocker, Merck Millipore 2170) in 0.1 M PB. The primary antibodies were diluted in 0.1 M PB, containing 5% (v/v) CB and 0.3% (v/v) Triton X-100 and applied for 12 h at 4 °C. For the negative controls, no primary antibodies were added. After incubation, slices were washed three times with 0.1 M PB containing 2% (v/v) CB. For secondary detection, the fluorescent labeled secondary antibodies were diluted in 0.1 M PB, containing 3% (v/v) CB and applied the antibodies for 1 h in the dark, followed by three times washing with 0.1 M PB. Cell nuclei were stained with Hoechst 33342 (5 µg/mL), which was applied for 10 min in the dark, followed by one washing step with 0.1 M PB. After mounting (Mountant Permafluor Thermo Scientific TA-030-FM), the slices were analyzed using a Leica SP8 confocal laser scanning microscope with the associated LAS X software (Leica, Wetzlar).

### ICC

ICC experiments were performed as IHC experiments, but instead of 0.1 M PB PBS^+^ was used. When ICC of transfected HEK293T cells was analyzed, Image J (version 2.0.0) was used.

### PLA

For the PLA experiments, Duolink InSitu Orange Starter Kit (Sigma-Aldrich DUO92102 and DUO92106) was used. Until the application of primary antibodies, the procedure followed the IHC and ICC procedures described above. PLA probes without primary antibodies served as a negative control. After incubation with primary antibodies, slices/cells were washed once with 0.1 M PB/PBS^+^. The following steps were carried out as described by the manufacturer with modifications after the last washing step as described in the manual with 0.01 wash buffer B. Before staining of cell nuclei and mounting, slices/cells were washed with 0.1 M PB/PBS^+^. Where applicable, Alexa488-NeuN antibody was applied for 2 h in 2% (v/v) CB in 0.1 M PB and slices were washed afterwards three times with 0.1 M PB. However, Alexa488-NeuN application impaired the intensity of the PLA signal. Cell nuclei were stained with Hoechst 33342 (5 µg/mL), which was applied for 10 min in the dark, followed by one washing step with 0.1 M PB/PBS^+^. After mounting (Mountant Permafluor Thermo Scientific TA-030-FM), the slices were analyzed using a Leica SP8 confocal laser scanning microscope (Leica, Wetzlar).

### Isolation of genomic DNA (gDNA), RNA, and UHPLC-QQQ-MS

Isolation of gDNA, RNA, and UHPLC-QQQ-MS experiments were performed according to a previously published protocol^45^.

### Synthesis of the Gdh inhibitor R162

Synthesis of the inhibitor R162 was done according to the literature^67,68^. Prior to use for the cell culture, the compound was purified via preparative HPLC (Nucleosil VP 250/10 C18 column Macherey Nagel, 100% MeCN for 10 min).

### Depolarization of hippocampal neurons followed by UHPLC-QQQ-MS analysis

#### Animals

Four- to five-week-old C57-BL6/J (Charles River, Sulzfeld, Germany) wild-type mice of both genders were used.

#### Hippocampal slices

Acute transverse hippocampal slices (400 μm thick) were prepared as described previously^69,70^. In brief, the brain was removed, the hippocampi of each hemisphere were dissected and cut using a MX-TS tissue slicer (Siskiyou Cooperation, OR). The slices were collected in an oxygenated (95% O_2_, 5% CO_2_) physiological solution (118 mM NaCl, 3 mM KCl, 1 mM NaH_2_PO_4_, 25 mM NaHCO_3_, 10 mM glucose, 1.5 mM CaCl_2_, 1 mM MgCl_2_, 0.1% (v/v) DMSO) at 37 °C until the hippocampi of all replicates were cut. Then the slices were distributed to three different conditions: oxygenated physiological solution, oxygenated 25 mM KCl solution (118 mM NaCl, 25 mM KCl, 1 mM NaH_2_PO_4_, 25 mM NaHCO_3_, 10 mM Glucose, 1.5 mM CaCl_2_, 1 mM MgCl_2_, 0.1% (v/v) DMSO) and oxygenated 25 mM KCl solution supplemented with 20 µM inhibitor R162 or 40 µM UK5099 (Sigma-Aldrich PZ0160-5MG). Six to ten slices were pooled for each replicate. After 6 h incubation time, the slices were transferred into reaction tubes, snap frozen in liquid nitrogen and stored at −80 °C until use. Isolation of gDNA, subsequent digest of gDNA, and analysis by UHPLC-QQQ-MS were performed according to a previously published protocol^45^.

We excluded mice from further analysis (hmdC quantification and gene expression) when *Npas4* expression was not upregulated in the depolarized hippocampal slices compared to the unstimulated control (one mouse out of eight for R162 experiment and two mice out of eight for UK5099 experiment) as this is an indicator that stimulation did not work, and the slices might be damaged.

### RT-qPCR

For cDNA synthesis, the RevertAid First Strand cDNA Synthesis Kit (Thermo Scientific K1621) was used according to the manufacturer’s instructions. One hundred ninety nanograms of total RNA was used for cDNA synthesis. Real-time quantitative PCR (RT-qPCR) was performed on StepOnePlus Real-Time PCR system (Applied Biosystems Thermo Fisher Scientific) using PowerUp^TM^ SYBR^TM^ Green qPCR Master Mix (Applied Biosystems Thermo Fisher Scientific A25742). *C*_T_ values of each sample were determined in technical duplicates by the StepOne^TM^ Software (Applied Biosystems Thermo Fisher Scientific) using the fast cycle protocol. The relative expression levels of target genes were then quantified from seven biological replicates (*n* = 7) according to Pfaffl et al.^71,72^.

The primers for RT-qPCR are listed in Supplementary Table 1 and the following primers have been published previously:

Npas4 (mouse)^73^

Bdnf total (mouse)^74^

### Metabolomics

Pre-weighed frozen hippocampal tissues from six mice (*n* = 6) per condition (physiological solution, 25 mM KCl, 25 mM KCl + R162) were homogenized using a bullet blender and five 1.4 mm stainless steel beads in a mixture of 1.05 ml ice-cold methanol/chloroform 1:2 (v/v). Following sonication of supernatants for 1 min, an additional 0.7 ml mixture of chloroform/LC grade water, 1:1 (v/v) were added, samples were vortexed for 20 s and incubated on wet ice for 30 min. Extracts were then centrifuged for 5 min at 16,000 × *g* and 4 °C and 0.6 ml from the upper aqueous phase were collected and dried under nitrogen gas. For NMR measurements, the dried extracts were dissolved in 0.25 ml 0.15 M phosphate buffer (pH 7.4) in deuterated water containing 0.04 mM sodium trimethylsilylpropionate-*d*_4_ (TSP) as internal standard. One 1D proton NMR was collected for each sample in a 14.1 T Bruker Avance II NMR under standardized conditions as described in Kostidis et al., 2017^40^. The NMR buffer was degassed, and NMR tubes were flushed with nitrogen to prevent oxidation. Metabolites were quantified in Chenomx NMR suit 8.4 (Chenomx Inc, Canada) and concentrations were corrected according to the tissue mass per sample. One mouse was completely excluded from further analysis as one sample out of the three conditions was lost during the sample preparation.

### Statistics and Reproducibility

#### Details about the statistical analysis

Statistical analysis, except for LFQ data processing as described above, was performed using GraphPad Prism (version 8.0.0 or higher) and only measurements from biological independent samples were considered.

Figure 3d:

For each biologically independent sample, hmdC/dN, fdC/dN, and cadC/dN values were normalized to the GFP signal to balance different Tet3 expression levels between the GFP-Tet3 and the GFP-Tet3 + Gdh-FLAG expressing cells, resulting in hmdC/(dN*RFU), fdC/(dN*RFU) and cadC/(dN*RFU). Samples were grouped (columns: Group A GFP-Tet3, Group B GFP-Tet3 + Gdh-FLAG; rows: 10, 24, 48 h post transfection; *n* = 3 biologically independent samples for each condition). For analysis, multiple t-tests (one per row, all of them two-sided) were chosen with individual variances between the rows. Statistical significance was determined using Holm-Sidak method with alpha = 0.05 to correct for multiple comparisons.

hmdC/(dN*RFU): 10 h: *p* value 0.000026, *p*_adj_ value 0.000078

24 h: *p* value 0.002581, *p*_adj_ value 0.005156

48 h: *p* value 0.003545, *p*_adj_ value 0.005156

fdC/(dN*RFU):10 h: *p* value 0.022270, *p*_adj_ value 0.038038

24 h: p value 0.009375, *p*_adj_ value 0.027862

48 h: *p* value 0.019204, *p*_adj_ value 0.038038

cadC/(dN*RFU):10 h: - 24 h: *p* value 0.009375, *p*_adj_ value 0.030692

48 h: *p* value 0.019204, *p*_adj_ value 0.040533

Figure 4a:

Differences between hmdC and fdC levels of GFP-Tet3 + Gdh-FLAG and GFP-Tet3 + Gdh-FLAG-NES expressing cells were analyzed individually for each biological replicate by calculating the paired t-test (two-sided *p* value).

hmdC: paired *t* test *p* value 0.0468; correlation coefficient *r* of the pairing 0.9492 (*p* value 0.0068, significantly effective pairing).

fdC: paired *t* test *p* value 0.0081; correlation coefficient r of the pairing 0.9708 (*p* value 0.0030, significantly effective pairing).

Figure 4d–f:

For each mouse, the prepared hippocampal slices were distributed to three different conditions – (A) physiological buffer, (B) 25 mM KCl, (C) 25 mM KCl + Inhibitor (20 µM R162 or 40 µM UK5099).

Figure 4d:

hmdC level R162 experiment:

*n* = 7, RM one-way ANOVA with Geisser–Greenhouse correction *p* value 0.0052; matching *p* value (one-sided) <0.0001. Tukey’s multiple comparisons test with individual variances computed for each comparison: A/B *p* value 0.0136, A/C *p* value 0.9995, B/C *p* value 0.0037.

hmdC level UK5099 experiment:

*n* = 6, RM one-way ANOVA with Geisser–Greenhouse correction *p* value 0.0034; matching *p* value (one-sided) <0.0001. Tukey’s multiple comparisons test with individual variances computed for each comparison: A/B *p* value 0.0410, A/C p-value 0.0123, B/C p-value 0.0130.

Figure 4e:

αKG level (normalized to mg of tissue) R162 experiment *n* = 5, ordinary one-way ANOVA *p* value 0.0002 (Brown-Forsythe test *p* value 0.1512). Tukey’s multiple comparisons test A/B *p* value 0.0001, A/C *p* value 0.0279, B/C *p* value 0.0230.

αKG/succinate ratio R162 experiment: *n* = 5, ordinary one-way ANOVA p-value 0.0368 (Brown–Forsythe test p-value 0.7828). Tukey’s multiple comparisons test A/B *p* value 0.1215, A/C *p* value 0.7684, B/C *p* value 0.0364.

Figure 4f:

*Npas4* gene expression level R162 experiment normalized to the gene expression level of the unstimulated control: *n* = 7, unpaired t-test with Welch’s correction (*F* test *p* value 0.3443) *p* value (two-sided) 0.0131.

*Bdnf* gene expression level R162 experiment normalized to the gene expression level of the unstimulated control: *n* = 7, unpaired *t*-test with Welch’s correction (*F* test *p* value 0.0208) *p* value (two-sided) 0.0136.

Supplementary Fig. 5d:

For each sample (*n* = 3 biologically independent samples), hmdC/dN and fdC/dN values were normalized to the GFP signal to balance different Tet3 expression levels between the samples (GFP-Tet3 A, GFP-Tet3 + 4 mM DM-αKG B, GFP-Tet3 + Gdh-FLAG C, GFP-Tet3 + Gdh-FLAG-NES), resulting in hmdC/(dN*RFU) and fdC/(dN*RFU).

For hmdC, ordinary one-way ANOVA *p* value 0.0209 (Brown–Forsythe test *p* value 0.6166). Tukey’s multiple comparisons test A/B *p* value 0.6289, A/C *p* value 0.0103, A/D *p* value 0.1509.

For fdC, ordinary one-way ANOVA *p* value 0.0070 (Brown–Forsythe test *p* value 0.6725). Tukey’s multiple comparisons test A/B *p* value 0.9942, A/C *p* value 0.0052, A/D *p* value 0.3030.

#### Details about the reproducibility

Figure 1b:

The preparation of the nuclear lysate from mESCs (4d primed) and total murine brain (4–5 weeks old wt BL6/J) was performed from two biologically independent samples/animals. These preparations were subsequently analyzed by western blotting against Tet1, Tet2, Tet3, and histone H3. Figure 1b shows representative western blots of this experiment.

Figure 2d:

coIP of endogenous Tet3 and a corresponding control coIP (using a non-targeting anti-goat antibody) was performed twice using two biologically independent nuclear lysate preparations from total murine brain (three months old wt BL6/J) and analyzed by western blotting. Figure 2d shows one representative blot of this experiment.

Figure 2e:

Tet3-Gdh PLA was performed in total four times using coronar cryo-sections of snap-frozen adult mouse brain from two biologically independent animals. Figure 2e shows one representative PLA result.

Figure 3a:

IHC/IF in hippocampus (Tet3 and Gdh) was performed in total four times using coronar cryo-sections of snap-frozen adult mouse brain from two biologically independent animals. IHC/IF in liver (Tet3 and Gdh) was performed two times using coronar cryo-sections of snap-frozen adult mouse liver from one animal. Figure 3a shows one representative IHC result.

Figure 3b:

Cell fractionation preparation from total murine brain (4–5 weeks old wt BL6/J) was performed from four biologically independent animals. These preparations were subsequently analyzed by western blotting. Figure 3b shows one representative western blot of this experiment.

Figure 3d:

GFP-Tet3 + Gdh-FLAG PLA or GFP + Gdh-FLAG PLA were performed twice in two biologically independent samples. Figure 3d shows one representative PLA result.

Figure 4b:

ICC/IF in GFP-Tet3 + Gdh-FLAG or GFP + Gdh-FLAG expressing HEK293T cells was performed three times using three biologically independent samples. Figure 4b shows one representative ICC result.

Figure 4c:

ICC/IF in FLAG_3_-Tet3-T2A-Glud1 transfected or untransfected HEK293T cells was performed three times using three biologically independent samples. Figure 4c shows one representative ICC result.

Figure 4g:

Tet3-NPC PLA and Gdh-NPC PLA were performed twice using coronar cryo-sections of snap-frozen adult mouse brain from one animal. Figure 4g shows one representative PLA result.

### Reporting summary

Further information on research design is available in the Nature Research Reporting Summary linked to this article.

## Supplementary information

Supplementary Information

Reporting Summary

## Data Availability

All datasets generated and analyzed during the current study are available from the corresponding author on reasonable request. Source data are provided with this paper. Proteomics data accession: The mass spectrometry proteomics data have been deposited to the ProteomeXchange Consortium via the PRIDE partner repository with the dataset identifier PXD004518. The metabolomics data have been deposited to the MetaboLights repository^75^ with the dataset identifier MTBLS2852. Source data are provided with this paper.

## Source data

Source Data file
