# Supplementary material for: Redirected nuclear glutamate dehydrogenase supplies Tet3 with α-ketoglutarate in neurons
Source: Nat Commun. 2021 Jul 2;12:4100. doi: 10.1038/s41467-021-24353-9 (PMC8253819; doi:10.1038/s41467-021-24353-9)
Supplement: Supplementary file 1 — Supplementary Information [file 41467_2021_24353_MOESM1_ESM.pdf]

Supplementary Material

Redirected nuclear glutamate dehydrogenase supplies Tet3 with α-ketoglutarate in neurons

Franziska R. Traube, Dilara Özdemir, Hanife Sahin, Constanze Scheel, Andrea F. Glück, Anna S. Geserich, Sabine Oganesian, Sarantos Kostidis, Katharina Iwan, René Rahimoff, Grazia Giorgio, Markus Müller, Fabio Spada, Martin Biel, Jürgen Cox, Martin Giera, Stylianos Michalakis, Thomas Carell

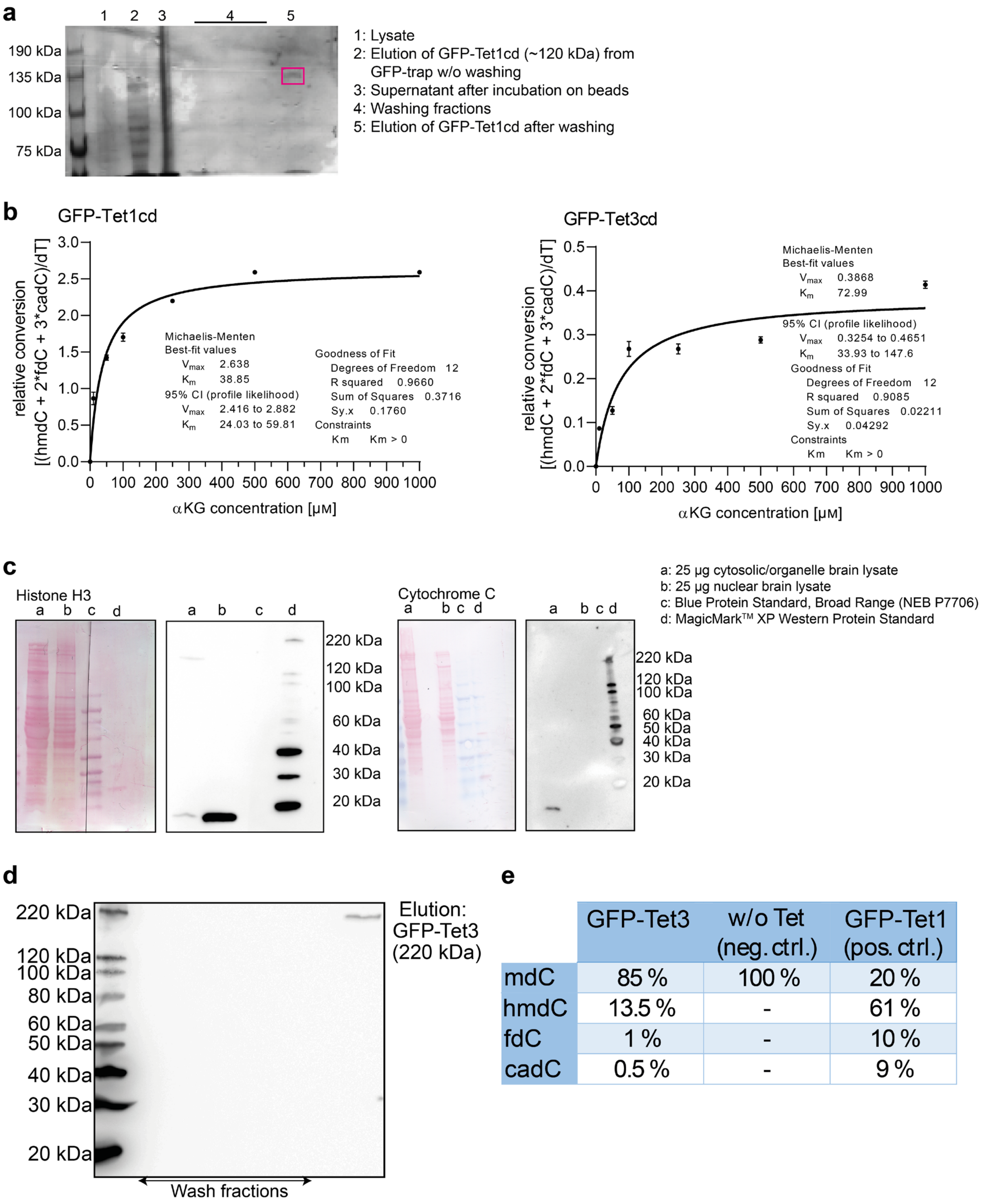

	GFP-Tet3	w/o Tet (neg. ctrl.)	GFP-Tet1 (pos. ctrl.)
mdC	85 %	100 %	20 %
hmdC	13.5 %	-	61 %
fdC	1 %	-	10 %
cadC	0.5 %	-	9 %

Supplementary Fig. 1: **Purification of overexpressed GFP-Tet fusion constructs, enzyme kinetics of GFP-Tet1 and GFP-Tet3 and subcellular prefractionation validation. a** Coomassie-stained SDS polyacrylamide gel showing different steps of GFP-Tet1cd purification of GFP nanobeads. The SDS-APGE was performed once. **b** Michaelis-Menten kinetics (n = 2 independent experiments) of GFP-Tet1cd and GFP-Tet3cd to determine the αKG concentration where half-maximal conversion rate is achieved. **c** Western blotting against histone H3 (15.4 kDa; nuclear marker) and cytochrome C (11.6 kDa; mitochondrial marker) confirmed that the nuclear brain extract is specifically enriched for nuclear proteins. Figure shows representative blots from one of four independent nuclear lysates, which were prepared from four biologically independent animals. **d** Western blot against GFP during purification of GFP-Tet3. The control western blot was performed once. **e** After purification, GFP-Tet3 activity was tested once using the described *in vitro* activity assay, which was analyzed by UHPLC-QQQ-MS. Source data are provided as a Source Data file.

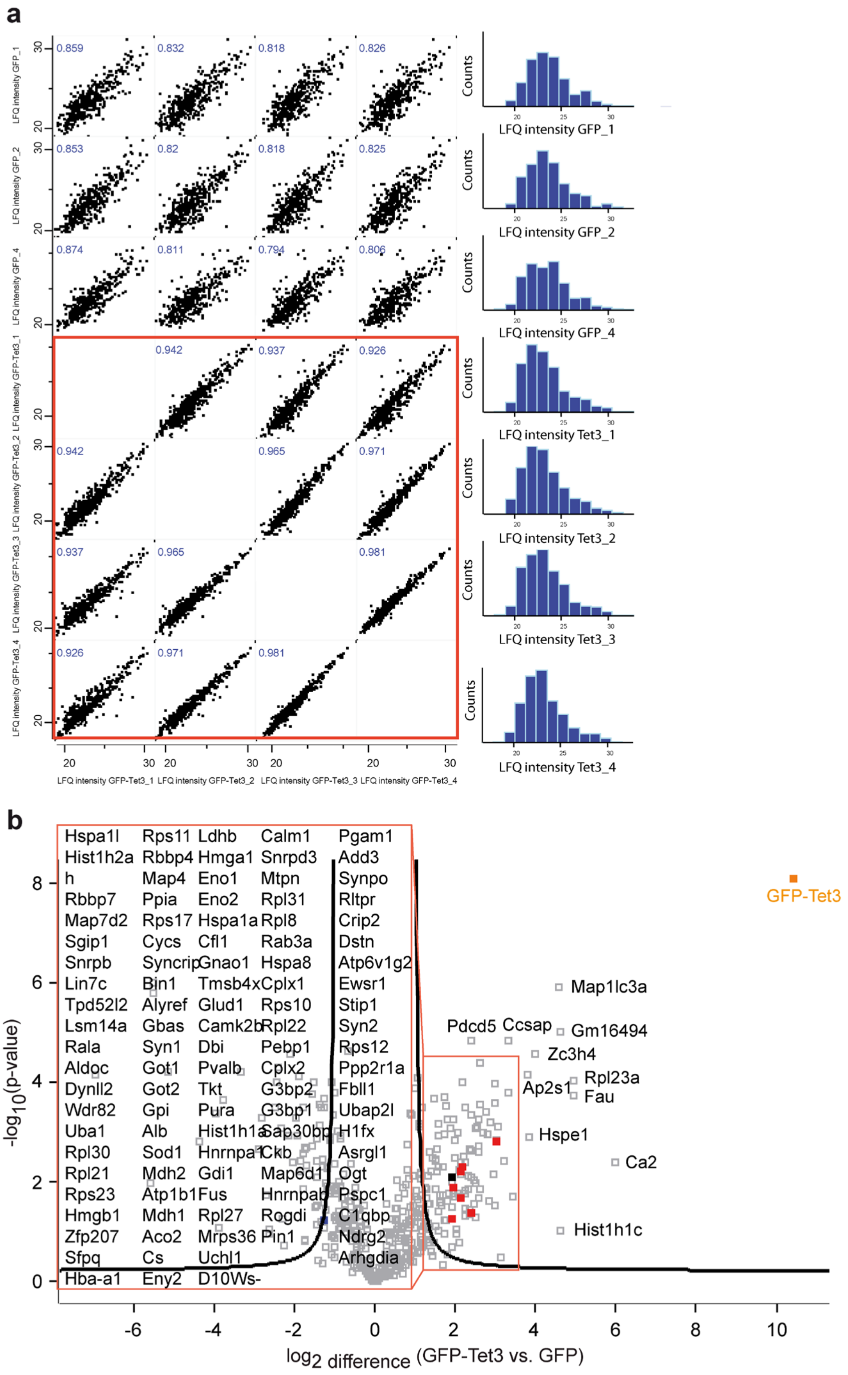

Supplementary Fig. 2: **LC-MS/MS analysis of Tet3-enriched coIP in murine brain. a** Multi scatter plot comparing the label-free quantification (LFQ) intensities from the four biological replicates of the GFP-Tet3 experiment with itself (red rectangle) and the pull downs using beads loaded with GFP. Blue numbers indicate the Pearson correlation. Histograms show counts of LFQ intensities of the data set. **b** Complete volcano plot of the interaction partners found in the coIP proteomics study in adult mouse brain for GFP-Tet3 (n = 4 biologically independent samples) (FDR = 0.05; s0 = 2). CoIP with GFP served as negative control (n = 3 biologically independent samples).

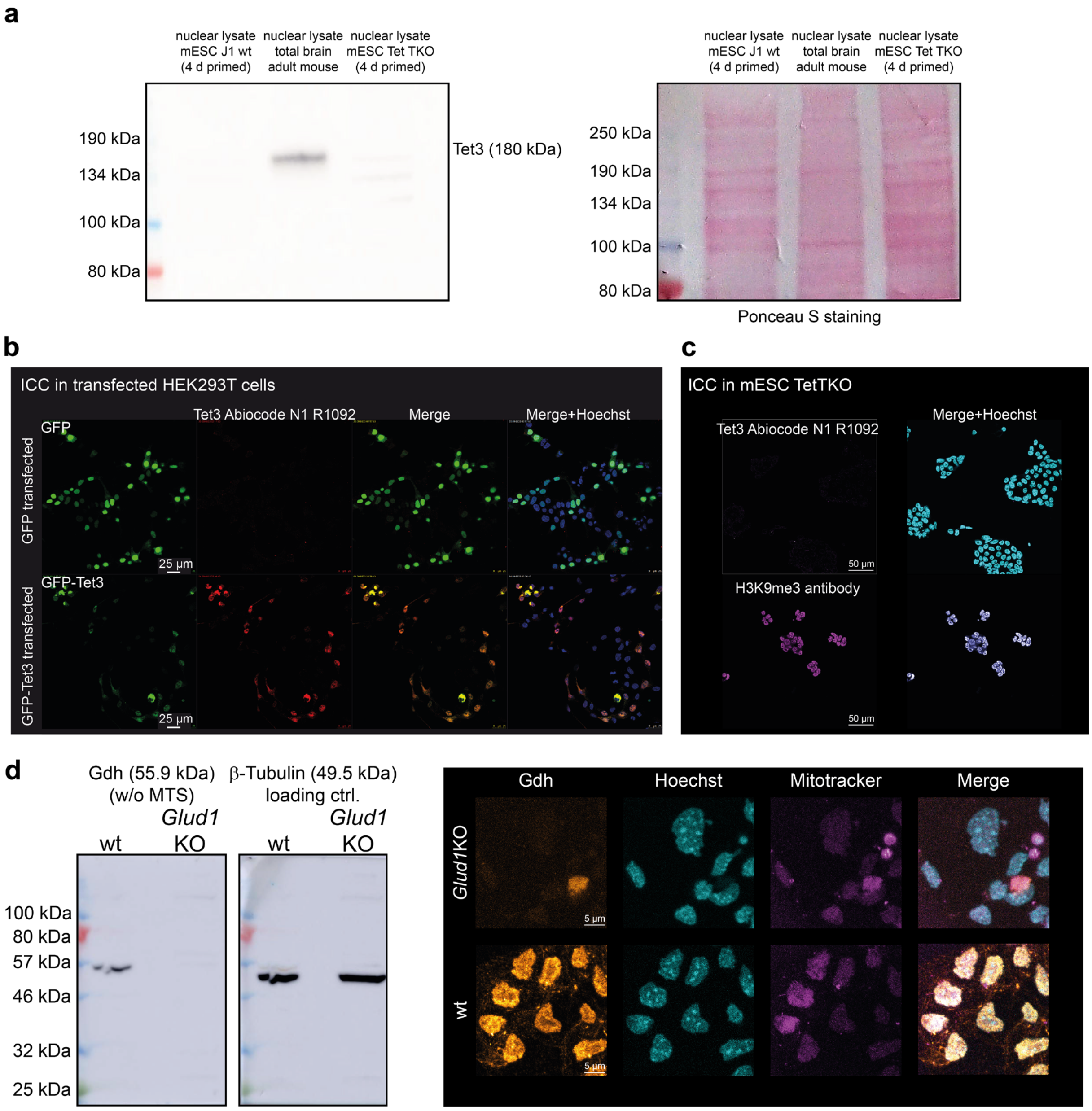

Supplementary Fig. 3: **Tet3 and Gdh antibody validation. a** Western blot of Tet3 in nuclear lysate from mESC J1wt, murine brain and mESC TET TKO and Ponceau S staining indicating equal protein load. **b** Validation of the used anti-Tet3 antibody using transfected HEK293T cells. The antibody showed no signal overlap with GFP in GFP-transfected HEK293T cells, but total signal overlap with GFP in GFP-Tet3 transfected HEK293T cells. **c** TET TKO mESC did not show a signal for Tet3 either when using this antibody. Histone modification H3K9me3 was tested in the same ICC as a positive control. **d** Validation of the used anti-Gdh antibody using haploid *Glud1*$^{-/}$ (mESC). Western blot and ICC confirmed the specifity of the antibody. β-tubulin was used as a loading control. **a** – **d** All panels show one representative result from two biologically independent samples. Source data are provided as a Source Data file.

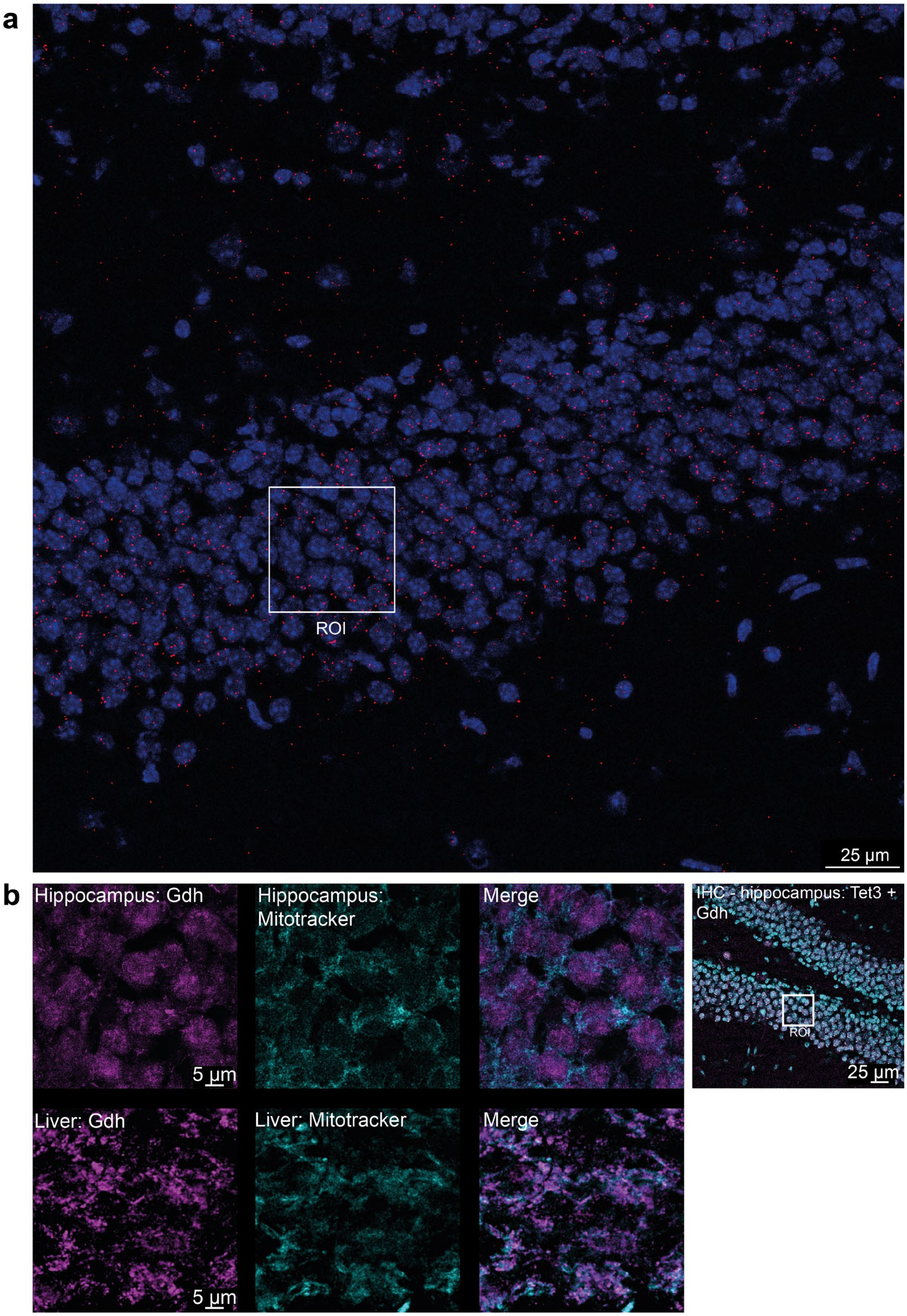

Supplementary Fig. 4: **PLA in hippocampus and ICC/IF in hippocampus and liver. a** PLA Tet3/Gdh in murine hippocampus with the region of interest (ROI) that marks the region that was chosen for the close-up image in Figure 1e. PLA signal is shown in red, nuclear staining (Hoechst) is shown in blue. **b** IHC of Figure 2a showing the corresponding mitotracker signal. Overview of the hippocampal regions for IHC of Tet3 and Gdh, in which ROI marks the region that was chosen for close-up image.

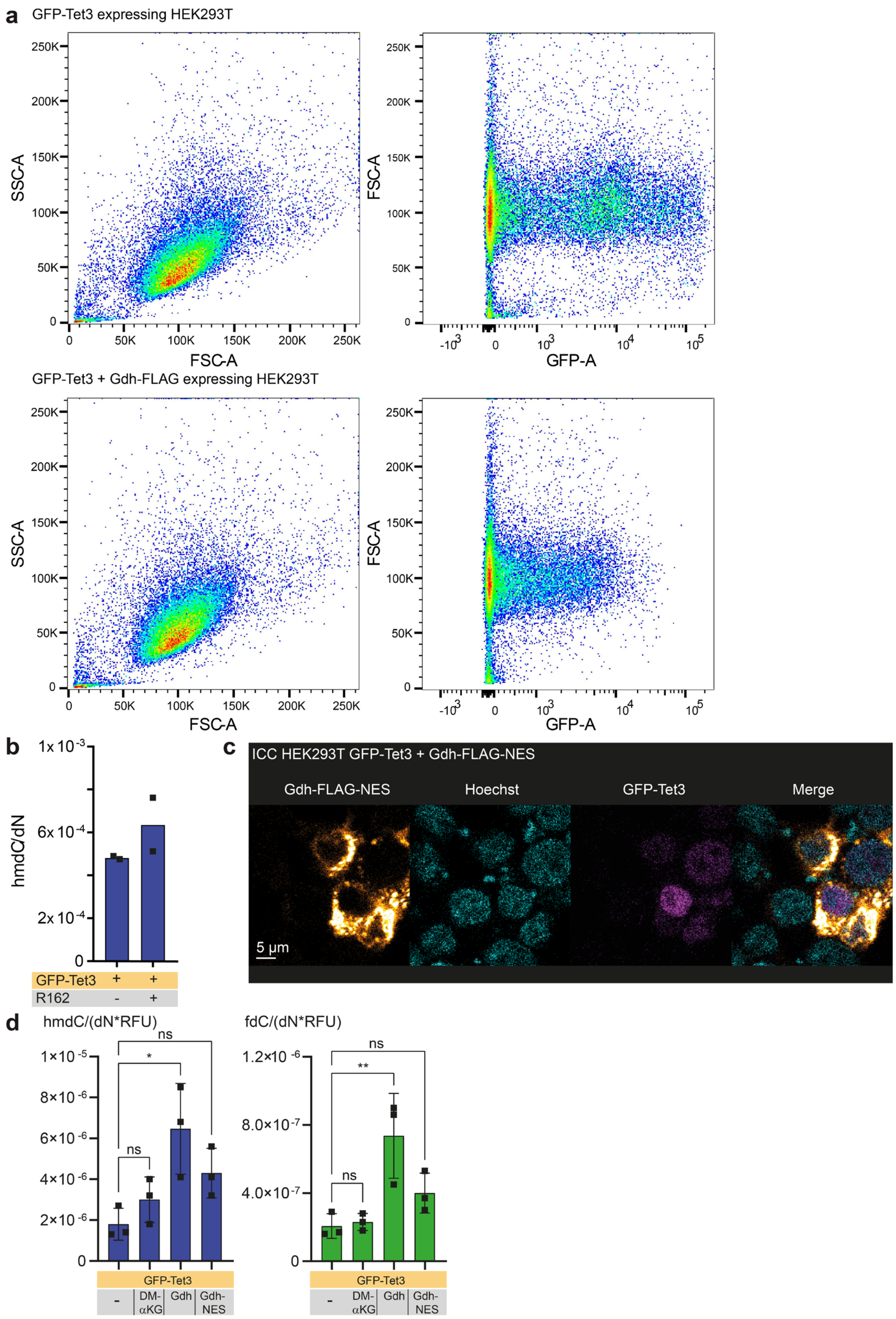

Supplementary Fig. 5: **FACS of GFP-Tet3 and GFP-Tet3 + Gdh-FLAG expressing HEK293T, R162 test on HEK293T and Gdh-FLAG-NES localization. a** Representative FACS plots of GFP-Tet3 and GFP-Tet3 + Gdh-FLAG expressing HEK293T showing that the GFP signal was more intense in the GFP-Tet3 only expressing cells, which indicates higher GFP-Tet3 levels. Therefore, the resulting hmdC, fdC and cadC levels were normalized to the mean of the GFP signal for each sample. **b** Gdh inhibitor R162 did not lower levels of hmdC in GFP-Tet3 expressing HEK293T (n = 2 independent samples, bars show mean). **c** Gdh-FLAG-NES + GFP-Tet3-transfected HEK293T do not show Gdh localization in the nucleus. The experiment was performed with three biologically independent samples and the graph shows one representative result. **d** Levels of hmdC and fdC 24 h after transfection of HEK293T (n = 3 biologically independent samples). Levels of mdC oxidation products were normalized to the GFP-signal of the cells (ordinary one-way ANOVA, $p_{adj} < 0.05$ (*), < 0.001 (**)). Bars show mean, error bars SD.

a

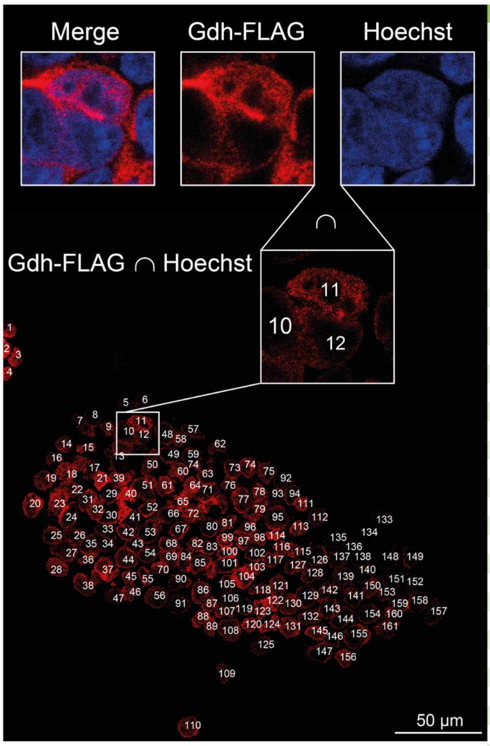

Cell Nr.	Mean	Mean>30	Cell Nr.	Mean	Mean>30	Cell Nr.	Mean	Mean>30	Cell Nr.	Mean	Mean>30
1	57.818	1	42	24.000		83	27.314		124	44.940	58
2	95.768	2	43	15.229		84	25.225		125	13.799	
3	66.690	3	44	28.252		85	26.904		126	8.081	
4	55.741	4	45	27.240		86	26.942		127	25.457	
5	7.710		46	39.609	18	87	38.653	36	128	21.061	
6	3.778		47	16.941		88	42.127	37	129	15.496	
7	10.863		48	11.649		89	41.710	38	130	30.709	59
8	3.541		49	14.000		90	10.120		131	43.049	60
9	22.918		50	22.420		91	6.014		132	21.719	
10	17.565		51	27.195		92	7.251		133	3.981	
11	48.705	5	52	33.724	19	93	8.906		134	1.697	
12	17.385		53	16.925		94	16.853		135	2.931	
13	26.781		54	18.873		95	16.846		136	5.242	
14	18.992		55	33.556	20	96	33.373	39	137	10.433	
15	23.450		56	19.893		97	43.169	40	138	6.358	
16	14.843		57	15.369		98	40.590	41	139	13.927	
17	27.751		58	36.316	21	99	70.788	42	140	8.548	
18	58.579	6	59	14.018		100	57.693	43	141	16.710	
19	33.141	7	60	42.625	22	101	13.549		142	22.253	
20	44.983	8	61	51.190	23	102	17.576		143	17.672	
21	83.030	9	62	19.611		103	65.114	44	144	13.539	
22	36.572	10	63	14.847		104	67.286	45	145	39.268	61
23	69.166	11	64	75.033	24	105	20.477		146	14.563	
24	20.393		65	61.961	25	106	12.904		147	14.221	
25	27.052		66	12.094		107	33.258	46	148	3.000	
26	24.704		67	20.128		108	25.531		149	9.613	
27	23.746		68	30.774	26	109	12.871		150	24.421	
28	38.750	12	69	31.852	27	110	34.976	47	151	8.707	
29	11.673		70	40.417	28	111	52.907	48	152	6.649	
30	21.636		71	18.788		112	20.405		153	14.261	
31	26.483		72	57.331	29	113	47.895	49	154	11.018	
32	48.907	13	73	27.558		114	88.783	50	155	16.538	
33	14.375		74	44.340	30	115	38.455	51	156	31.652	62
34	14.103		75	26.690		116	44.215	52	157	9.579	
35	18.382		76	21.180		117	29.234		158	9.705	
36	28.604		77	34.593	31	118	30.418	53	159	9.939	
37	65.294	14	78	47.927	32	119	11.148		160	23.568	
38	33.803	15	79	41.163	33	120	52.574	54	161	19928	
39	56.326	16	80	11.630		121	35.155	55			
40	104.994	17	81	37.053	34	122	79.326	56			
41	21.119		82	42.173	35	123	54.708	57			

b HEK293T

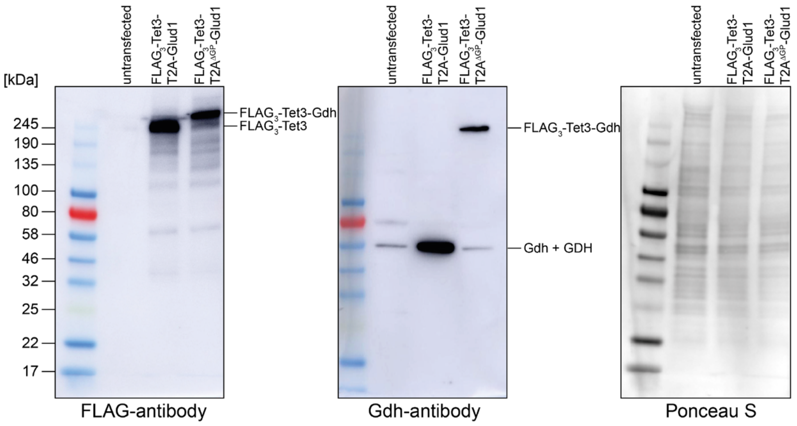

c

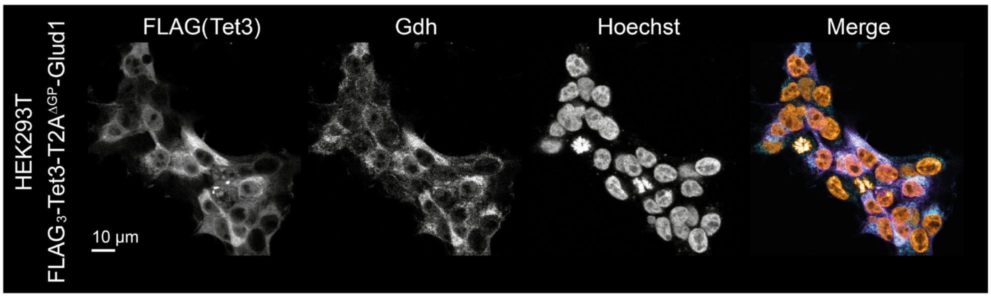

Supplementary Fig. 6: **Ectopic co-expression of Tet3 and Gdh in HEK293T. a** ICC of GFP-Tet3 + Gdh-FLAG expressing HEK293T cells. Calculation of the brightness value per cell of the overlap of Gdh-FLAG and Hoechst from the ICC using image J. A mean brightness value above 30 was considered as a nuclear signal. Approximately 40% of the cells show Gdh-FLAG signal in the nucleus, when co-expressed with GFP-Tet3. Graph shows one representative result of three biologically independent samples. **b** Western blots detecting FLAG-tagged Tet3 and Gdh or GDH of HEK293T total lysate (20 µg) of untransfected cells and cells that were either transfected with a bicistronic vector coding for $FLAG_3$-Tet3-T2A-Glud1 that results in $FLAG_3$-Tet3 and Gdh or a $FLAG_3$-Tet3-$T2A^{\Delta GP}$-Glud1 vector that results in a FLAG3-Tet3-Gdh fusion protein. Ponceau S staining indicates equal protein amounts. **c** ICC of HEK293T expressing the FLAG3-Tet3-Gdh fusion construct showing the fusion protein remains in the cytosol and does not enter the nucleus. **b** and **c** Graphs show one representative result of three biologically independent samples. Source data are provided as a Source Data file.

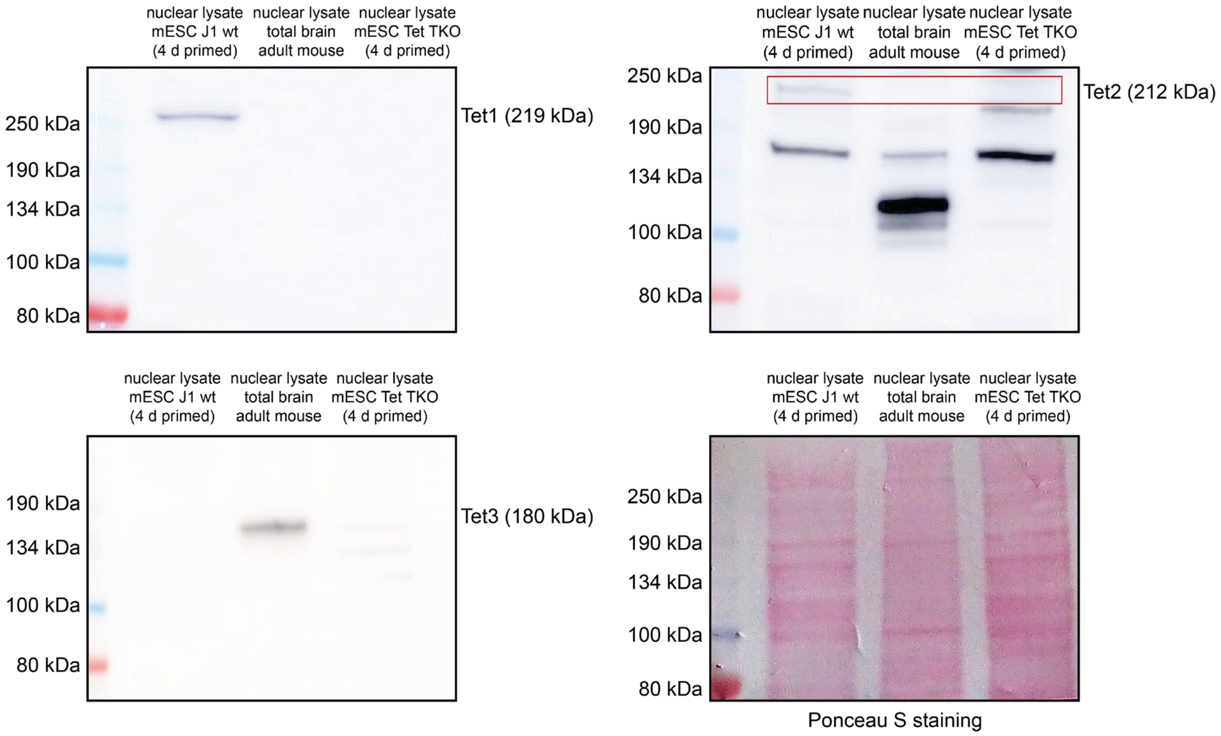

Supplementary Fig. 7: **Tet antibody validation.** Western blots of Tet1, Tet2 and Tet3 in nuclear lysate from mESC J1wt, murine brain and mESC TET TKO and Ponceau S staining indicating equal protein load. Source data are provided as a Source Data file.

Supplementary Table 1: Primers used for RT-qPCR.

Gene	Forward primer (5' – 3')	Reverse primer (5' – 3')	References
Alas (mouse)	TCG CCG ATG CCC ATT CTT ATC	GGC CCC AAC TTC CAT CAT CT	
Npas4 (mouse)	CTG CAT CTA CAC TCG CAA GG	GCC ACA ATG TCT TCA AGC TCT	[73]
Bdnf total (mouse)	GCC TTT GGA GCC TCC TCT	CTG TCA CAC ACG CTC AGC TC	[74]
